# Effect of Bi_2_MoO_6_ Morphology on Adsorption and Visible-Light-Driven Degradation of 2,4-Dichlorophenoxyacetic Acid

**DOI:** 10.3390/molecules29143255

**Published:** 2024-07-10

**Authors:** Thi Thanh Hoa Duong, Shuoping Ding, Michael Sebek, Henrik Lund, Stephan Bartling, Tim Peppel, Thanh Son Le, Norbert Steinfeldt

**Affiliations:** 1 Leibniz Institute for Catalysis e.V. (LIKAT), Albert-Einstein-Street 29a, 18059 Rostock, Germany; hoa.duong@catalysis.de (T.T.H.D.); shuoping.ding@catalysis.de (S.D.); michael.sebek@catalysis.de (M.S.); henrik.lund@catalysis.de (H.L.); stephan.bartling@catalysis.de (S.B.); tim.peppel@catalysis.de (T.P.); 2 Faculty of Chemistry, VNU University of Science, Hanoi 100000, Vietnam; sonlt@vnu.edu.vn

**Keywords:** 2,4-dichlorophenoxyacetic acid, Bi_2_MoO_6_, photodegradation, adsorption, hydrogen peroxide

## Abstract

The development of highly efficient and stable visible-light-driven photocatalysts for the removal of herbicide 2,4-dichlorophenoxyacetic acid (2,4-D) from water is still a challenge. In this work, Bi_2_MoO_6_ (BMO) materials with different morphology were successfully prepared via a simple hydrothermal method by altering the solvent. The morphology of the BMO material is mainly influenced by the solvent used in the synthesis (H_2_O, ethanol, and ethylene glycol or their mixtures) and to a lesser extent by subsequent thermal annealing. BMO with aggregated spheres and nanoplate-like structures hydrothermally synthesized in ethylene glycol (EG) and subsequently calcined at 400 °C (BMO-400 (EG)) showed the highest adsorption capacity and photocatalytic activity compared to other synthesized morphologies. Complete degradation of 2,4-D on BMO upon irradiation with a blue light-emitting diode (LED, λ_max_ = 467 nm) was reached within 150 min, resulting in 2,4-dichlorophenol (2,4-DCP) as the main degradation product. Holes (h^+^) and superoxide radicals (⋅O_2_^−^) are assumed to be the reactive species observed for the rapid conversion of 2,4-D to 2,4-DCP. The addition of H_2_O_2_ to the reaction mixture not only accelerates the degradation of 2,4-DCP but also significantly reduces the total organic carbon (TOC) content, indicating that hydroxyl radicals are crucial for the rapid mineralization of 2,4-D. Under optimal conditions, the TOC value was reduced by 84.5% within 180 min using BMO-400 (EG) and H_2_O_2_. The improved degradation performance of BMO-400 (EG) can be attributed to its particular morphology leading to lower charge transfer resistance, higher electron–hole separation, and larger specific surface area.

## 1. Introduction

In recent years, 2,4-dichlorophenoxyacetic acid (2,4-D) has become one of the most widely used herbicides for the control of broadleaf weeds and plant growth regulators for wheat, oats, corn, barley, rye, and sugar cane in agriculture all over the world [1]. As reported, the presence of the herbicide 2,4-D in aquatic environments is toxic to the ecosystems and human health, particularly its ability to induce cytogenetic damage to human lymphocytes, irreversible eye damage, nephrotoxicity, and hepatotoxicity [2]. To date, many traditional methods have been proposed to treat herbicide pollution, such as the application of membrane bioreactor technology [3], bacterial strains [4], biological degradation [5], and electrochemical methods [6,7]. However, these traditional processes still suffer from major drawbacks, such as high operational costs, high energy consumption, low efficiency, and long reaction times.

Photocatalysis was considered an alternative technique to overcome the disadvantages mentioned above. In this method, charge carriers (electrons and holes) are generated by irradiating the photocatalyst with light. The charge carriers generated can react directly with a substrate or contribute to the formation of reactive oxygen species such as hydroxyl radicals (·OH) and superoxide radicals (·O_2_^−^). Photocatalysis has already been successfully applied in energy demands (e.g., water splitting [8,9] and CO_2_ reduction [10]), chemical synthesis [11], and environmental remediation [12,13,14,15,16,17,18]. Various photocatalysts have been used to degrade 2,4-D in aqueous solutions, such as the anatase phase of TiO_2_ nanocrystals anchored on SBA-15 mesoporous [15,19], transition metals (Co, Cu, Fe, or Ni) deposited on TiO_2_ [20], ZnAlFe LDH [21], or Fe_2_O_3_/CeO_2_/Ag composites [22], respectively. All these materials have been shown to be active only when irradiated with ultraviolet (UV) light. Under these conditions, 2,4-D was degraded by hydroxyl radicals (⋅OH) or direct oxidation of 2,4-D with holes (h^+^) [23]. The synthesis of photocatalysts, in which catalytically active species are already generated upon irradiation under visible light, is of great importance, as the ultraviolet component of sunlight accounts for only 4% [24]. Until now, the photocatalytic degradation of 2,4-D and especially its mineralization under irradiation with visible light has remained a challenge [25,26,27]. As active species for the degradation of 2,4-D by visible light, holes (h^+^) as well as superoxide radicals (⋅O_2_^−^) were identified [25]. On the other hand, the mechanism for 2,4-D mineralization under such conditions is still unclear. Moreover, highly toxic and potentially carcinogenic 2,4-dichlorophenol (2,4-DCP) is often formed as the main degradation product under UV [27] and visible light irradiation [25,27]. 

Among numerous materials, bismuth-based semiconductors are known as active materials in the field of photocatalysis and energy conversion due to their intrinsic physical and chemical properties [28,29,30]. Bi_2_MoO_6_ (BMO) consists of alternating (MoO_4_)^2−^ perovskite, and fluorite-like (Bi_2_O_2_)^2+^ layers have already been widely applied in the field of photocatalysis, e.g., photocatalytic water splitting, energy storage, and supercapacitors [24,31,32,33,34,35,36,37]. BMO is a non-toxic material, which shows a band-gap energy suitable for harvesting visible light [38,39,40,41]. Both BMO-based composites, as well as pure the BMO phase, have been applied for the photocatalytic degradation of dye, antibiotic, and herbicide contaminants [42]. Pd/g-C_3_N_4_/Bi_2_MoO_6_ can degrade Rhodamine B (RhB) under visible light irradiation [43]. The Bi_2_MoO_6_/Ag_3_PO_4_ composite was applied to the degradation of MB under LED light illumination (λ = 410 nm) [44]. A Z-scheme Bi_2_MoO_6_/CNTs/g-C_3_N_4_ composite was employed for enhancing the debromination and degradation of 2,4-dibromphenol [45]. The Ag_2_O-Bi_2_MoO_6_ p- n heterojunction photocatalyst showed excellent photocatalytic activity in the removal of phenol and RhB [46], and atomic-level 2D/2D defective Bi_2_MoO_6_/g-C_3_N_4_ effectively degraded ciprofloxacin [47]. However, the synthesis of composite materials is often time-consuming, and/or relatively expensive starting materials have to be used. 

Improved charge carrier separation and enhanced charge carrier transportation can also be achieved by controlling the morphology of the pure BMO phase. BMO can be synthesized by a solid-state reaction [48], co-precipitation [49], the sol–gel method [50], or in a hydrothermal process. While the first three methods require a calcination step for BMO crystallization, in the hydrothermal process, crystalline BMO is already formed in the reaction solution. The morphology of the formed BMO particles in the hydrothermal process can be affected by the used solvent and the synthesis parameters. BMO microspheres composed of nanosheets synthesized in ethylene glycol (EG) showed better performance than commercial P25 titania in the RhB degradation under Xe-light irradiation in the presence of H_2_O_2_ [51]. An EG solvent can also be used to form small BMO nanosheets, which have shown optimal performance for visible-light-driven Rhodamine B (RhB) degradation [52]. BMO nanosheets also showed significantly faster photocatalytic degradation of methylene blue (MB) under visible light irradiation than BMO nanorods, which were synthesized in nitric acid solution in the presence of ammonia [53]. BMO nanoplatelets can be formed in a mixture of EG and H_2_O. Under visible light irradiation, degradation of the antibiotic ofloxacin and the dye Congo red was 1.3- and 1.65-fold faster compared to commercially available P25 titanium oxide [49]. The photocatalytic activity of hydrothermally synthesized BMO can be further improved by heat treatment [28]. The annealing process can induce an upward shift of the valence band maximum and conduction band minimum edge in BMO. 

Based on the above considerations, it is highly interesting to investigate the catalytic performance of BMO in degrading the herbicide 2,4-D under visible light irradiation. With regards to this, Bi_2_MoO_6_ materials with different morphologies were synthesized via a facile hydrothermal process using H_2_O, ethanol (EtOH), ethylene glycol (EG), and their mixture as solvents. The materials were characterized by complementary methods and tested in the degradation of 2,4-D under blue light irradiation. Preliminary experiments were carried out to study the adsorption of 2,4-D on BMO materials. Noteworthily, the adsorption capacity of 2,4-D on BMO synthesized in EG can be significantly improved by thermal treatment. Furthermore, the influence of BMO morphology on the photocatalytic degradation and mineralization of 2,4-D was investigated, including the addition of hydrogen peroxide. The role of the BMO photocatalyst in pollutant degradation in the presence of H_2_O_2_ under blue LED light irradiation was proposed.

## 2. Results and Discussion

### 2.1. Catalyst Characterization

#### 2.1.1. Structural Characteristics

**Figure 1:**
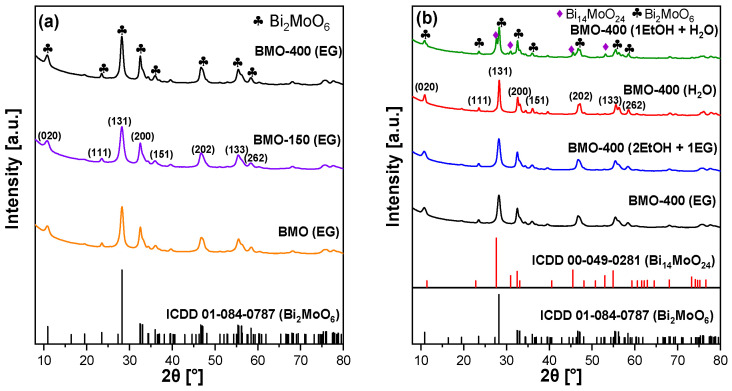
XRD powder pattern of BMO samples (**a**) synthesized in ethylene glycol and after thermal annealing and (**b**) BMO synthesized using different solvents and after calcination in static air.

XRD powder patterns of BMO samples are presented in Figure 1. Figure 1a compares XRD patterns of BMO material synthesized in EG with and without thermal post-treatment. The main reflections centered at 2*θ* = 10.7, 28.1, 32.5, 34.3, 35.9, 46.8, 55.5, and 58.4° correspond to the (0 2 0), (1 3 1), (2 0 0), (0 6 0), (1 5 1), (2 0 2), (1 3 3), and (2 6 2) lattice planes of the orthorhombic Bi_2_MoO_6_ phase (ICDD 01-084-0787) [54,55,56]. Thermal annealing at 400 °C increases the average primary crystallite size from 6.7 nm to 10.9 nm (see Table 1). The pure BMO phase was also obtained in H_2_O or in a mixture with EG and EtOH as the synthesis solvent (Figure S4). Material that was synthesized in a mixture of EtOH and H_2_O shows, in addition to diffraction peaks of the orthorhombic Bi_2_MoO_6_ phase, additional reflections of Bi_14_MoO_24_ (ICDD 00-049-0281) [57]. The process of its formation is still unclear. A tiny amount of Bi(NO_3_)_3_·5H_2_O might be achieved to form an amorphous or nano-grain-sized hydrated bismuth oxide precursor inside the ethanol/H_2_O mixture. This precursor or Bi_2_O_3_ obtained from this precursor might further react with Bi_2_MoO_6_ to form crystalline B_14_MoO_24_. A similar reaction was previously proposed to occur during thermal treatment [57].

The phase composition was not influenced by calcination at 400 °C, as shown in Figure 1b. As already observed for the sample synthesized in EG, the smaller line width of the reflections indicates that calcination mainly influences the crystallinity. The average crystallite size of heat-treated samples synthesized in the presence of water was higher than that of samples obtained in the presence of EG (Table 1).

**Figure 2:**
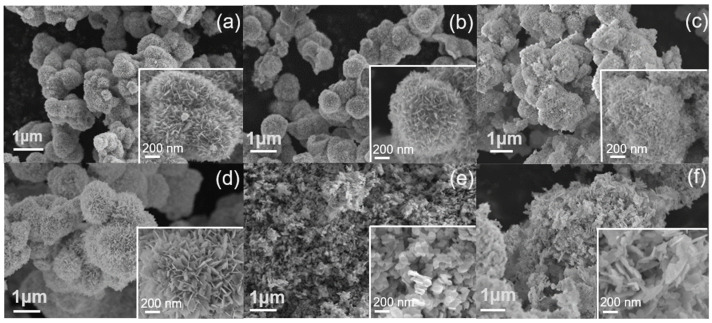
SEM images of (**a**) BMO (EG), (**b**) BMO-150 (EG), (**c**) BMO-400 (EG), (**d**) BMO-400 (2 EtOH + 1 EG), (**e**) BMO-400 (H_2_O), and (**f**) BMO-400 (1 EtOH + 2 H_2_O).

The morphologies of the photocatalysts were investigated using scanning electron microscopy (SEM). BMO (EG) and BMO-150 (EG) (Figure 2a,b) exhibit three-dimensional (3-D) hollow microspheres with single diameters ranging from 0.7 to 1.0 μm, which are composed of two-dimensional (2-D) nanosheets. When the annealing temperature was increased to 400 °C, the aggregated spheres were sintered together to form nanoplate-like structures (Figure 2c). The annealed BMO-400 (2 EtOH + 1 EG) displays flower-like aggregated spheres, but larger than those that were synthesized in pure EG. The flowers also consist of nanosheets (inset of Figure 2d). When BMO was synthesized in H_2_O and heat-treated, disordered nanoplates with an average diameter of 1.8 μm were observed (Figure 2e). Disordered nanoplates were also found in calcinated BMO material that was synthesized by the hydrothermal process when H_2_O with EtOH was used as solvents. SEM images of BMOs synthesized in solvent mixtures (2 EtOH + 1 EG, H_2_O, and 1 EtOH + 2 H_2_O) before thermal annealing are presented in Figure S5. The images reveal structures similar to the annealed counterparts, which is in agreement with previously reported results [28]. The morphology of BMO depends mainly on the solvent used for the hydrothermal process, and thermal annealing up to 400 °C affects the morphology but only to a moderate extent.

To gain further insights into the molecular structures of the photocatalysts, FT-IR spectra were recorded (Figure S6). For all samples, a set of bands was observed between 400 and 900 cm^−1^ that are characteristic of Bi_2_MoO_6_ [58]. Bands at 798 and 840 cm^−1^ were previously attributed to the Mo-O symmetric and asymmetric stretching vibration modes in the MoO_6_ octahedral structure [53,59]. The bands located at 720 and 558 cm^−1^ can be ascribed to the bending vibration of the MoO_6_ octahedral structure [59,60,61]. For the BMO (EG) and BMO-150 (EG) samples, an additional band was detected at 1580 cm^−1^ of the O-H stretching vibration of adsorbed ethylene glycol, which disappears after treatment at 400 °C. 

#### 2.1.2. Thermal Stability and Textural Properties

Because the BMO materials were synthesized in organic solvents, their surface might be covered by organic residues, thus TGA measurements were performed. The weight loss of freshly synthesized BMO (EG) in the N_2_ atmosphere from 30 °C to 600 °C is shown in Figure S7a. Between 50 and 150 °C, the weight loss (9.3%) is likely due to the desorption of water and organic components with low boiling points. The observed weight loss of approximately 7% between 160 and 500 °C can be attributed to the decomposition of EG residues because already calcined material (400 °C) lost only 0.6% weight. All calcined samples (Figure S7b) in TGA measurements had a slight weight loss that agrees well with the FT-IR results, which illustrates no bands originating from carbonaceous species after heat treatment. Therefore, the annealing temperature of 400 °C appears to be sufficient to almost completely remove organic residues from the synthesis process.

The BET surface area (SA) and pore structure of heat-treated BMO materials were determined by N_2_ sorption. The BET surface area of the materials originating directly from the hydrothermal process was not estimated, as the sorption measurement requires thermal pretreatment. The adsorption–desorption isotherms of annealed materials (400 °C) are depicted in Figure S8. The isotherms can be classified as type IV with an H3 hysteresis loop, which indicates the presence of mesopores [54]. The BET surface area and the pore volume (PV) were influenced by the solvent applied for BMO synthesis (Table 1) and ranged from 7.0 to 33.4 m^2^·g^−1^ and 0.04 to 0.21 cm^3^·g^−1^. The BMO sample with the largest BET surface area and pore volume was synthesized in EG. 

#### 2.1.3. Near-Surface Composition

**Figure 3:**
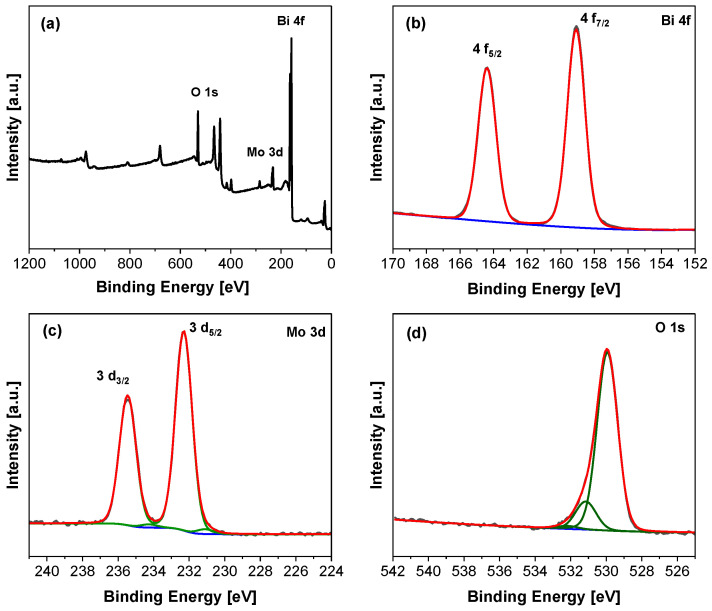
(**a**) Survey XPS spectrum and high-resolution spectra of BMO-400 (EG) of Bi 4f (**b**), Mo 3d (**c**), and O 1s (**d**).

The near-surface elemental composition and the oxidation state of surface elements were analyzed using X-ray photoelectron spectroscopy (XPS). As shown in Figure 3a, Bi, Mo, and O elements were found to have strong characteristic peaks in the survey spectrum of the BMO-400 (EG) sample. The high-resolution XP spectrum of Bi 4f (Figure 3b) shows peaks located at 164.3 eV and 159.1 eV, which are ascribed to Bi 4*f*_5/2_ and Bi 4*f*_7/2_ and correspond to the +3 oxidation state of Bi [62]. Moreover, the Mo 3d spectrum presents peaks at binding energies of 235.5 eV and 232.2 eV that are attributed to Mo 3d_3/2_ and Mo 3d_5/2_ of Mo^6+^, respectively [63,64]. A second small Mo 3d doublet is observed at 234.1 eV and 231.4 eV, which is characteristic of Mo^5+^ [65]. In addition, the high-resolution signal of O 1s shows peaks at 531.1 eV and 529.9 eV (Figure 3d), which can be attributed to bridging hydroxyl species and lattice oxygen [63], respectively. 

#### 2.1.4. Optical, Electronic, and Photo-Electrochemical Characterization

**Figure 4:**
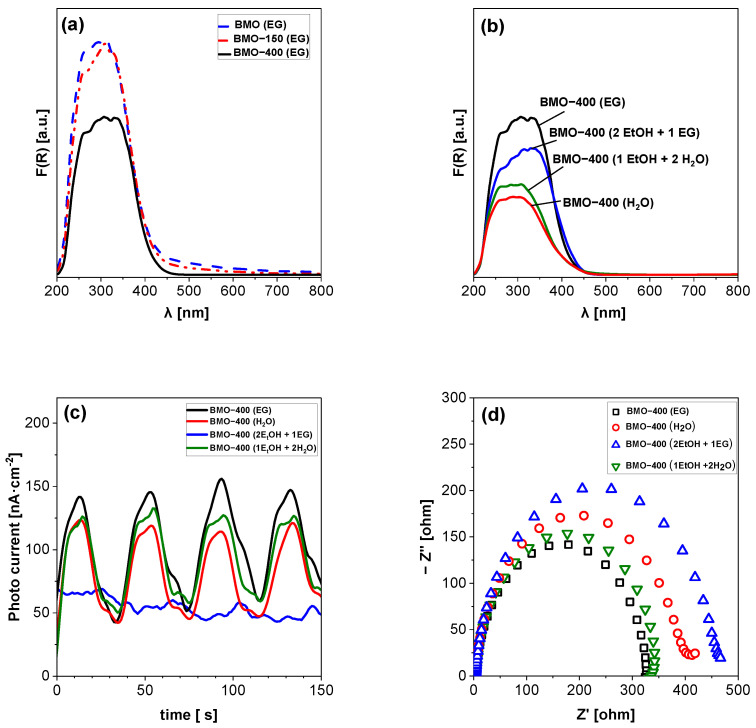
UV-Vis diffuse reflectance spectra of (**a**) BMO (EG) after its thermal annealing, (**b**) BMO synthesized with different solvents after thermal annealing at 400 °C, (**c**) photocurrent responses, and (**d**) EIS Nyquist plots of calcined BMO samples obtained under irradiation with visible light (λ = 430 nm).

UV-vis diffuse reflectance spectroscopy (DRS) was applied to study light absorption. As shown in Figure 4a,b, all materials absorb light in the UV and visible light region up to 500 nm. The band gap energies were calculated using the Kubelka–Munk function [66] and are summarized in Table 1. For all BMO samples, almost identical band gaps are estimated with values of around 2.75 eV. 

Photoluminescence (PL) measurements are often used to investigate the recombination of the generated charge carriers. As shown in Figure S9, all samples show a relatively low PL intensity, but the PL intensity of BMO-400 (EG) was the lowest of all, indicating that the carrier recombination in this sample is the lowest.

Photoelectrochemical properties were investigated via the photocurrent response and electrochemical impedance spectroscopy (EIS). As presented in Figure S10a, BMO-400 (EG) exhibits a higher photocurrent density than BMO (EG), which indicates that after calcination under irradiation, a higher number of charge carriers can be generated or generated charges can be more effectively separated in the calcined samples. When comparing the photocurrent density of the four calcined samples (Figure 4c), BMO-400 (EG) shows a slightly higher current density than BMO-400 (1 EtOH + 2 H_2_O) and BMO-400 (H_2_O). Surprisingly, the measured photocurrent density of BMO-400 (2 EtOH + 1 EG) is much lower than that of the other three materials. EIS was employed to explore the ability of the samples to transfer charge carriers under blue LED light irradiation. Figure S10b gives evidence that the arc radius for BMO-400 (EG) is much smaller than that of BMO (EG), indicating a lower charge transfer resistance and improved electron–hole separation in the calcined sample. When comparing the arc radius of the different samples under visible light irradiation (Figure 4d), the same order was found as that for photocurrent density. BMO-400 (EG) shows the smallest arc radius, followed by BMO-400 (1 EtOH + 2 H_2_O) and BMO-400 (H_2_O). Mott–Schottky (MS) plots are presented in Figure S11. The positive slope of the MS curve implies that BMO is an n-type semiconductor. For this type, the flat band potential is close to the conduction band potential. The MS plots were used to calculate the conduction band minimum edge (CBM). The valence band maximum (VBM) edges were estimated by using the band gap. Both values are summarized in Table S1. The VBM edge varies between 2.11 and 2.25 V, and the CBM edges are between −0.63 and −0.50 V. The edge positions are within the range given in the literature for VBM (between 1.98 and 2.44 V) and CBM (between −0.29 and −0.64 V) [39,40,41,55,62,67,68]. No clear correlation could be derived between the sample morphology and the CB and VB edge positions. On the other hand, the obtained results indicate that thermal annealing only affects the edge positions to a minor extent, which differs from previously reported results [28].

### 2.2. Adsorption of 2,4-D

**Figure 5:**
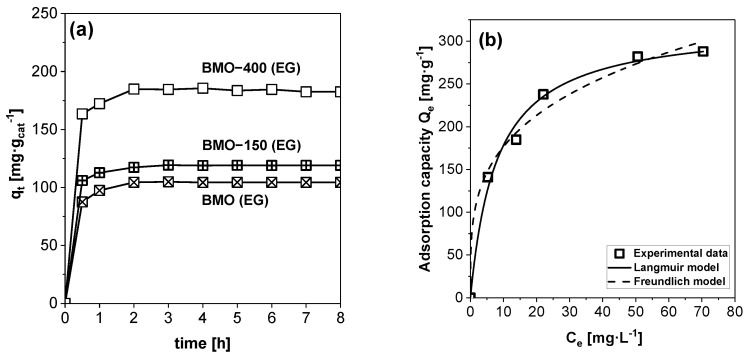
(**a**) Plot of adsorbed 2,4-D amount versus time on BMO (EG) with and without thermal annealing, and (**b**) plot of 2,4-D adsorption capacity versus 2,4-D equilibrium concentration for BMO-400 (EG) and a fit using Langmuir and Freundlich adsorption isotherm models.

The influence of contact time on the adsorption of 2,4-D on BMO samples prepared in EG was studied at 25 °C with an initial 2,4-D concentration of 20 mg·L^−1^ (Figure 5a). Within the first 30 min, the adsorption process was relatively fast, which can be explained by the large number of free adsorption sites for 2,4-D molecules. Between 30 and 120 min, a slower adsorption rate was observed, and after 120 min, the adsorption/desorption equilibrium of 2,4-D was achieved on all three materials. The capacity of the material to adsorb 2,4-D increased from 100 mg·g_cat_^−1^ (as-synthesized) to 184.8 mg·g_cat_^−1^ when it was heat-treated at 400 °C. It has already been shown that organic residues can be removed from the BMO surface by the annealing process (Figure S7a), which should provide more accessible surface sites for the adsorption of 2,4-D molecules. The thermally treated BMO materials that were synthesized in other solvents as EG showed lower adsorption capacities of around 100 mg·g_cat_^−1^ (Figure S12). Another reason for the higher adsorption capacity of BMO-400 (EG) could be its higher surface carbon content (approximately 10%, Table S7). This might contribute to the fact that the surface of BMO-400 (EG) is more hydrophilic than that of BMO-400 (H_2_O), as shown by the lower contact angle (Figure S13). To exclude the possibility that the observed differences in adsorption are caused by differences in the BMO surface charge between the annealed samples, their zeta potential was measured (Figure S14). All annealed materials show a positive surface charge at the pH value obtained after the dissolution of 2,4-D in the aqueous phase (pH = 4.96). The differences in surface charge between the individual BMO samples are relatively small. A correlation between the surface charge and adsorption capacity could not be established, indicating that surface charge is not the main reason for the higher adsorption capacity of BMO-400 (EG).

To determine the maximum loading capacity, adsorption experiments were performed at different 2,4-D concentrations with BMO-400 (EG) because of its high adsorption capacity. Figure 5b presents the relationship between the obtained equilibrium concentration of 2,4-D (C_e_) and the equilibrium loading capacity (Q_e_). The results reveal that the adsorbed amount of the herbicide 2,4-D increases when increasing the initial concentration until a plateau is reached. The adsorption data of 2,4-D were fitted using both the Langmuir and Freundlich models. The adsorption isotherm obtained with the Langmuir model agrees better with the experimental data than the isotherm calculated with the Freundlich model (Table S2). Finally, the maximum adsorption capacity (Q_m_) of BMO-400 (EG) for 2,4-D was calculated with the Langmuir model and found to be relatively high (320.0 mg·g_cat_^−1^) when compared to capacities of other reported adsorbents (Table S3).

### 2.3. Photocatalytic Degradation of 2,4-D

#### 2.3.1. Results of Blue Light Irradiation

The results of the photocatalytic degradation of 2,4-D in the presence of BMO samples synthesized in EG are depicted in Figure S15. In the absence of the photocatalyst, 2,4-D was not degraded in the dark or under blue light irradiation (not illustrated). The heat-treated BMO sample degraded 2,4-D faster than the as-synthesized material (BMO-EG). When BMO-400 (EG) was used, approximately 70% of 2,4-D was converted after 30 min of illumination, while only 40% of 2,4-D was degraded on BMO (EG). As the main degradation product, 2,4-dichlorophenol (2,4-DCP) was detected, and its formation (mmol·L^−1^) is plotted as a function of the illumination time in Figure S15b.

**Figure 6:**
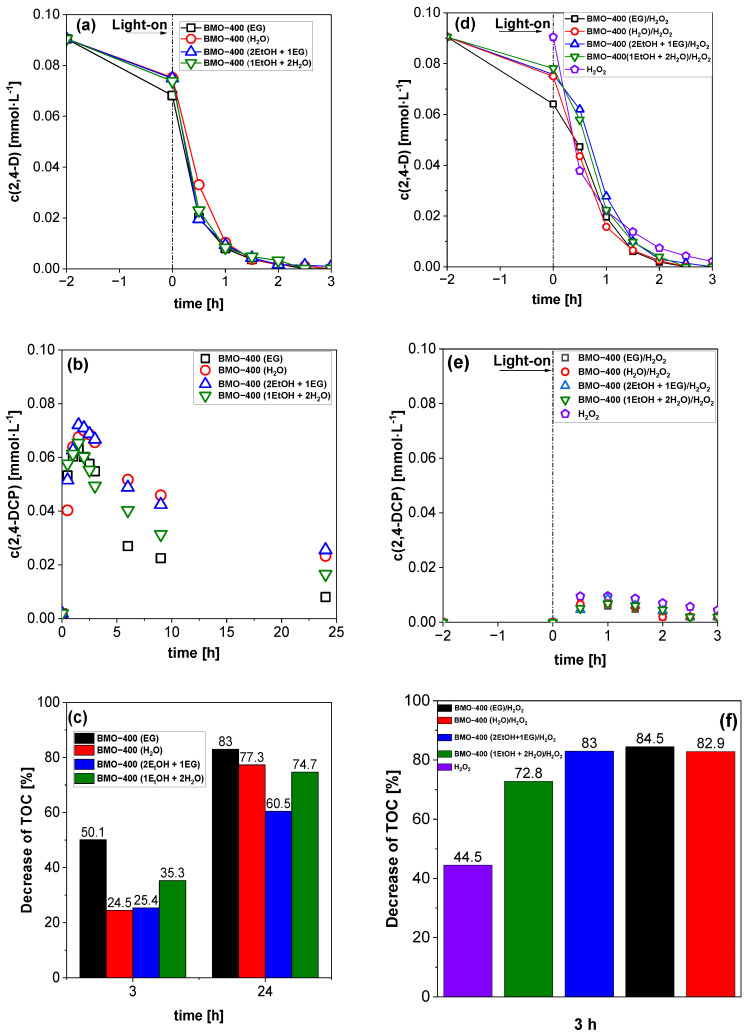
(**a**) Plot of 2,4-D concentration versus time for BMO photocatalysts synthesized in different solvents and thermal annealed at 400 °C, (**b**) plot of 2,4-dichlorophenol (2,4-DCP) concentration versus time, (**c**) evolution of TOC decrease, (**d**) plot of 2,4-D concentration versus time for BMO photocatalysts in presence of H_2_O_2_ (V(30% H_2_O_2_) = 1 mL), (**e**) plot of 2,4-DCP concentration versus time in presence of H_2_O_2_, and (**f**) TOC decrease in presence of H_2_O_2_ (m_cat_ = 10 mg, C_(2,4-D)_ = 20 mg·L^−1^, V = 30 mL, blue LED (λ_max_ = 467 nm), T = 25 °C).

Only minor differences in the herbicide degradation were observed for BMO samples synthesized in different solvents and not thermally treated (Figure S16). The photodegradation of 2,4-D was noticeably faster when annealed BMO materials (400 °C) were applied (Figure 6a). The reasons for this could be a higher number of active surface sites, better crystallinity, and reduced charge carrier recombination. The fastest rate of 2,4-D degradation was determined in the presence of BMO-400 (H_2_O). Figure S17 depicts the pseudo-first-order model that was used to demonstrate the kinetic behaviors of 2,4-D degradation over different materials, and Table S4 lists the corresponding fitted data. The values for the rate constants obtained with BMO are in the same order of magnitude as those obtained with other photocatalysts (Table S5). As shown in Figure 6b, within 2 h of light irradiation, 2,4-D was converted mainly into 2,4-dichlorophenol (2,4-DCP) via cleavage of acetic acid. The concentration of 2,4-DCP decreases slowly under blue light irradiation, indicating that 2,4-DCP is harder to degrade than 2,4-D under the applied test conditions. The fastest transformation of 2,4-DCP was observed in the presence of BMO-400 (EG), followed by BMO-400 (1 EtOH+ 2 H_2_O) (Figure 6b). In an additional experiment, 2,4-DCP was applied as a pollutant using the same reaction parameters as for the studies with 2,4-D. In a blank test without a catalyst, 16.5% of 2,4-DCP was degraded under 3 h of blue light irradiation. However, in the presence of 10 mg BMO-400 (EG), approximately 52% of 2,4-DCP was converted. This result indicates that BMO is also catalytically involved in the photodegradation of 2,4-DCP.

The amount of total organic carbon (TOC) in the irradiated reaction solutions was measured at specific time intervals to evaluate the efficiency of mineralization. When BMO-400 (EG) was used as the photocatalyst, less than 50% of TOC was detected in the solution after 3 h of irradiation (Figure 6c). The significant decrease in TOC when using BMO-400 (EG) is the sum of 2,4-D adsorption and its photocatalytic degradation, with 2,4-DCP as the main intermediate. When comparing the TOC removal results after 24 h, BMO-400 (EG) again shows the lowest TOC value.

**Figure 7:**
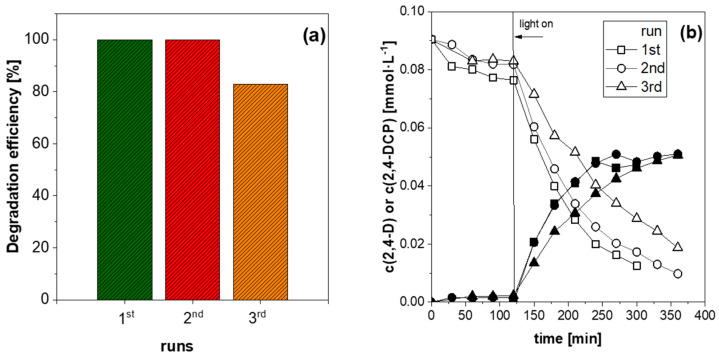
(**a**) Recycling runs in the photocatalytic degradation of 2,4-D with BMO-400 (EG) in small batch reactor and (**b**) plot of 2,4-D and 2,4-DCP concentration versus time in flow operation under recirculation (open symbols—2,4-D, filled symbols—2,4-DCP).

In further studies, the photocatalytic activity of BMO-400 (EG) was determined in three consecutive runs (Figure 7a). Prior to each run, the used catalyst was collected by centrifugation and washed carefully with deionized water and ethanol several times. The degradation efficiency remained constant at 100% for two reaction cycles but dropped to 83% in the third run. XPS analysis of BMO-400 (EG) after the third run and 24 h of the photocatalytic reaction showed a lower surface content of the elements Bi, Mo, and O and significantly higher carbon content compared to the unused BMO material (Table S7). It was therefore assumed that carbon-containing species had formed on the material surface, which deactivated the photocatalyst by blocking the active sites. The same deactivation behavior was observed when BMO-400 (EG) was deposited on an alumina mesh and tested in an irradiated micro-photoreactor under a continuous flow of recirculated 2,4-D reaction solution. Three flow experiments were carried out in sequence, and it was found that the adsorption and degradation of 2,4-D decreased from one experiment to the next (Figure 7b). Here, the difference in activity between the second and third tests was greater than that between the first and second tests, which is consistent with the results of the batch experiments. 

#### 2.3.2. Influence of H_2_O_2_

As Figure 6b shows, 2,4-D was converted to 2,4-DCP relatively quickly under irradiation with all BMO catalysts, while the further conversion of 2,4-DCP required a relatively long time. It is suspected that the formed superoxide radicals (·O_2_^−^) and holes (h^+^) are incapable of effectively degrading 2,4-DCP. To overcome this, hydrogen peroxide (H_2_O_2_), as an environmental oxidant, was added to the reaction suspension after achieving equilibrium in adsorption and desorption. It is known that H_2_O_2_ can be decomposed to produce hydroxyl radicals (⋅OH) already under irradiation without the need for a photocatalyst [69]. The amount of hydroxyl radicals formed by the photolysis of H_2_O_2_ depends on the potency of the light (wavelength and energy density) and the H_2_O_2_ concentration [70]. The results of degradation experiments in the presence of H_2_O_2_ are presented in Figure 6d–f. Upon 3 h of irradiation, 2,4-D was nearly completely converted but only by H_2_O_2_ (Figure 6d). Without light irradiation, 8.2% of 2,4-D was converted with H_2_O_2_ in 3 h. In the simultaneous presence of the photocatalyst and H_2_O_2_, the 2,4-D degradation rate was similar to that of when the BMO photocatalyst was used alone, but the amount of 2,4-DCP was significantly lower (Figure 6e). Its concentration in the irradiated suspension strongly depends on the H_2_O_2_ concentration used (compare results in Figure 6e and Figure S18b). 

Moreover, it was found that the TOC content of an irradiated 2,4-D solution was significantly lower when BMO was used as a photocatalyst together with H_2_O_2_. In contrast, a higher concentration of organic carbon was measured in the solution after photolysis when only H_2_O_2_ was applied (Figure 6f). Based on these results, it can be concluded that BMO plays an active role as a photocatalyst in the degradation process of 2,4-D. In the presence of the photocatalyst, H_2_O_2_ can not only be decomposed into hydroxyl radicals by light but it can also react with electrons in the BMO conduction band, also leading to the formation of reactive oxygen species like hydroxyl radicals [51]. The photolysis of H_2_O_2_ reduced the TOC content within 3 h by 44.5%, but 84.5% of the organic carbon was mineralized when H_2_O_2_ was used in excess together with BMO photocatalysts (Figure 6f). Even if the H_2_O_2_ concentration in the catalyst-assisted degradation process was reduced 1000-fold, the mineralization efficiency was still higher than that of H_2_O_2_ photolysis using a high H_2_O_2_ concentration (Figure S18c). The reason for the faster mineralization of 2,4-D in the presence of BMO could be due to the enhanced formation rate of hydroxyl ions and/or the contribution of photogenerated charge carriers (electrons and holes) to the degradation process after overcoming the 2,4-DCP barrier.

#### 2.3.3. Proposed Photocatalytic Degradation Mechanism

**Figure 8:**
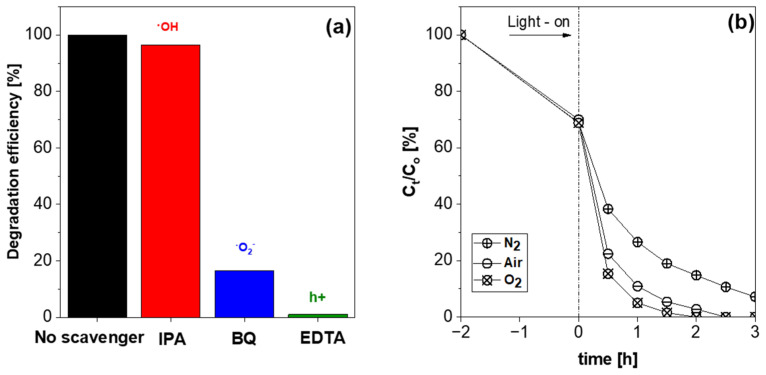
(**a**) Effect of scavenger on the 2,4-D degradation using blue LED light in the presence of BMO-400 (EG) after 180 min (m_cat_ = 10 mg, C_(2,4-D)_ = 20 mg·L^−1^, *n*(scavenger) = 0.15 mmol; (**b**) plot of relative 2,4-D concentration versus irradiation time using different gas atmospheres.

To explore the role of active species in the 2,4-D photodegradation process, experiments were performed using benzoquinone (BQ), isopropyl alcohol (IPA), and ethylenediaminetetraacetic acid (EDTA) as scavengers for superoxide radicals (⋅O_2_^−^), hydroxyl radicals (⋅OH), and holes (h^+^), respectively (Figure 8a). When IPA was added to the solution, the photodegradation efficiency of BMO-400 (EG) only slightly decreased, indicating that ⋅OH plays a minor role in the photocatalytic conversion of 2,4-D to 2,4-DCP as the main product after 3 h of blue light irradiation. When BQ was used as the scavenger, the 2,4-D degradation efficiency was significantly reduced, implying that ⋅O_2_^−^ was involved in the observed photodegradation of 2,4-D. The photocatalytic activity was mostly inhibited when EDTA was added, indicating that 2,4-DCP was likely formed by the reaction of 2,4-D with holes. Further experiments were carried out under a pure N_2_ and O_2_ atmosphere. The results presented in Figure 8b show that 2,4-D was decomposed slightly faster when O_2_ was introduced in the reaction solution instead of air, and the degradation was suppressed under N_2_ atmosphere. It was also observed that the concentration of 2,4-DCP, which is the major degradation product of 2,4-D, was significantly lower in the reaction mixture under inert N_2_ than in air or a pure O_2_ atmosphere (Figure S19b). This result indicates that the rate of 2,4-D degradation is influenced by the content of dissolved oxygen in the reaction mixture.

**Figure 9:**
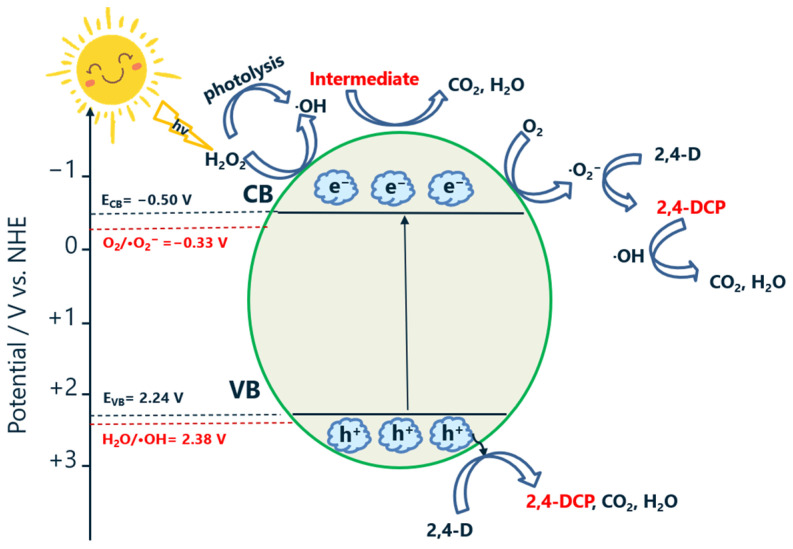
Scheme of proposed photocatalytic mechanism for 2,4-D degradation on BMO-400 (EG) under visible light irradiation and in the presence of H_2_O_2_.

Based on the presented results, a mechanism for the photocatalytic degradation of 2,4-D by BMO-400 (EG) was proposed in Figure 9. When the target material BMO-400 (EG) is irradiated by blue light, charge carriers (electrons and holes) are generated. The electrons accumulated in the conduction band (CB) are able to transform dissolved oxygen into superoxide radicals (⋅O_2_^−^). This process is thermodynamically allowed because the CBM edge of BMO-400 (EG) (−0.50 V versus NHE) is more negative than E (O_2_/⋅O_2_^−^ = −0.33 V versus NHE) [71]. Both superoxide radicals as well as the remaining holes in the VB can react with adsorbed 2,4-D, mainly forming 2,4-DCP. The degradation of the intermediate 2,4-DCP is assumed to occur slowly via the reaction with hydroxyl radicals. The energy of the VBM edge of BMO-400 (EG) (2.24 V) is lower than the standard potential of H_2_O/⋅OH (2.38 V versus NHE) [72] suggesting that the h^+^ in the VB cannot oxidize H_2_O molecules to generate ⋅OH, which is consistent with the results of the trapping experiment (Figure 8a). Therefore, a low amount of hydroxyl radicals might be formed under irradiation from the decomposition of H_2_O_2_ formed in situ. Such formation was previously suggested by the reaction of ⋅O_2_^−^ with protons (H^+^) and electrons from the CB [73]. To investigate whether ⋅OH was generated during the photocatalytic reaction, terephthalic acid (TPA) was used as a probe molecule. TPA can readily react with ⋅OH to produce the highly fluorescent product 2-hydroxyterephthalic acid [74]. As shown in Figure S20, the samples collected under blue light irradiation show photoluminescence (PL) resulting from the formation of 2-hydroxyterephthalic acid. The intermediates obtained from the reaction of 2,4-DCP and ⋅OH are rapidly degraded further by the main reactive species, holes, and superoxide radicals, eventually forming CO_2_ and water. 

If H_2_O_2_ is added to the 2,4-D solution, hydroxyl radicals are formed by H_2_O_2_ photolysis, which degrades 2,4-D and, in particular, 2,4-DCP in a relatively short time. The TOC content can be further reduced if the irradiated solution contains both the BMO photocatalyst and H_2_O_2_. The higher number of hydroxyl radicals in the presence of the photocatalyst is attributed to the reaction of H_2_O_2_ with the light-generated electrons in the BMO conduction band. In addition, the charge carriers generated in the BMO under irradiation could also be involved in the degradation process of the intermediates that occur.

## 3. Materials and Methods

### 3.1. Materials

Bi(NO_3_)_3_·5H_2_O (≥98.0%) was purchased from Chempur (Karlsruhe, Germany) and Na_2_MoO_4_·2H_2_O (≥99.5%) from Sigma (Setagaya City, Tokyo). Ethylene glycol (EG, ≥99.5%) was obtained from Carl Roth (Karlsruhe, Germany), isopropanol (IPA, ≥99.5%) from ACROS (Fukuoka, Japan), ethanol (EtOH, ≥99.8%) from Fisher Chemical (Hampton, VA, USA), and H_2_O_2_ (30 wt.%) from VWR (Radnor, PA, USA). 2,4-D (≥98.0%) and 2,4-DCP (≥98.0%) were purchased from Sigma-Aldrich (St. Louis, MO, USA). Sodium hydroxide (NaOH, ≥98.0%) and Terephthalic acid (≥98.0%) were obtained from Merck (Darmstadt, Germany). 

All chemicals and reagents were used as received from commercial suppliers without further purification. Deionized water was applied in the experiments. 

### 3.2. Synthesis of Bi_2_MoO_6_

The Bi_2_MoO_6_ materials were synthesized by a facile hydrothermal process following a protocol in the literature with slight modifications [53]. Using the typical procedure, 3.78 mmol (1.834 g) of Bi(NO_3_)_3_·5H_2_O and 1.89 mmol (0.457 g) of Na_2_MoO_4_·2H_2_O were mixed and 30 mL of the solvent (H_2_O, ethylene glycol, ethanol/water = 1 v/2 v, ethanol/ethylene glycol = 2 v/1 v) was added, respectively. The mixture was stirred for 30 min at an ambient temperature and transferred into a 45 mL Teflon-lined stainless-steel autoclave. The hydrothermal process was carried out at 160 °C for 20 h, and afterward, the autoclave was left to cool down naturally to room temperature. The resulting powders were collected by centrifugation (8000 rpm, 10 min), washed several times with deionized water and ethanol, and dried in an oven at 80 °C overnight. The obtained powders were calcined for 2 h in an air atmosphere at 400 °C with a heating rate of 5 K·min^−1^, respectively. In addition, a sample that was synthesized in ethylene glycol was calcined at 150 °C. The final powder products were labeled as BMO-150 (EG), BMO-400 (EG), BMO-400 (H_2_O), BMO-400 (1 EtOH + 2 H_2_O), and BMO-400 (2 EtOH+ 1 EG), respectively. 

### 3.3. Characterization 

The X-ray diffraction patterns were measured with an Xpert Pro diffractometer (Panalytical, Almelo, The Netherlands) using a scanning rate of 0.05°/s and monochromatized Cu Ka radiation. The primary crystallite size (D) was obtained by using the Scherrer equation:

(1) $$D=\frac{k\lambda }{\beta Cos\theta }$$

Here, *k* is the Scherrer constant (0.89), λ is the wavelength of Cu Kα radiation (0.154 nm), β is the width evaluated at mid-height of the intense diffraction peak, and θ is Bragg’s diffraction angle.

The XPS (X-ray Photoelectron Spectroscopy) measurements were performed on an ESCALAB 220iXL (Thermo Fisher Scientific, Waltham, MA, USA) with monochromated Al Kα radiation (E = 1486.6 eV). Samples were prepared on a stainless-steel holder with conductive double-sided adhesive carbon tape. The measurements are performed with charge compensation using a flood electron system combining low-energy electrons and Ar^+^ ions (p_Ar_ = 1 × 10^−7^ mbar). The electron binding energies are referenced as the C 1s core level of carbon at 284.8 eV (C-C and C-H bonds). For quantitative analysis, the peaks were deconvoluted with Gaussian–Lorentzian curves using the software Unifit 2023. The peak areas were normalized by the transmission function of the spectrometer and the element-specific sensitivity factor of Scofield.

Fourier transform infrared spectroscopy (FT-IR, Nicolet 330, Thermo Fisher Scientific, Waltham, MA, USA) was performed to determine the specific functional groups on the surface of samples under ambient conditions using KBr as the background. 

Light absorption was determined by UV-Vis diffuse reflectance spectroscopy (UV-Vis DRS) with a Carry-5000 (Agilent, Santa Clara, CA, USA) spectrophotometer from 200 to 800 nm with BaSO_4_ as the reference. 

The BET surface areas and porosities of the samples were obtained by N_2_ adsorption at −196 °C using a Micromeritics ASAP 2020 (Micromeritics, Norcross, GA, USA) instrument and calculated by the Brunauer–Emmett–Teller (BET) and Barrett–Joyner–Halenda (BJH) methods, respectively. Prior to the analysis, the samples were degassed at 350 °C for 5 h. 

Thermogravimetric analyses (TGA) and differential scanning calorimetry measurements (DSC) were performed in corundum crucibles in the temperature range of 25 to 600 °C with a heating rate of 10 K min^−1^ in a nitrogen atmosphere simultaneously on a NETZSCH STA 449 F5 Jupiter device (Netzsch, Selb, Germany). 

The SEM micrographs were recorded using a Merlin VP compact device (Zeiss, Oberkochen, Germany). 

The photoluminescence (PL) spectra were recorded using a Varian Cary Eclipse Fluorescence Spectrometer (Agilent Technologies, Mulgrave, Australia) with a Xenon lamp as an excitation source at an excitation wavelength of 370 nm. 

### 3.4. Electrochemical Characterization

The photoelectrochemical experiment was performed on a Zennium electrochemical workstation equipped with a PP211 CIMPS system (Zahner, Kronach, Germany) with typical three-electrode cells. The counter-electrodes were Pt wires, while the reference electrodes were saturated Ag/AgCl (3 M). The electrolyte was Na_2_SO_4_ (0.5 M). The working electrode was prepared using a coating method. Briefly, 10 mg of the photocatalyst was dispersed in the mixture of 100 µL of Nafion solution (5 wt.%) and 900 μL of isopropyl alcohol under ultrasound for 10 min, then the dispersion was dropped on a FTO glass with an active area of 1.5 × 1.5 cm^2^ and dried in the air. The transient photocurrent response was measured in a light on–off process with a pulse of 20 s. A 430 nm LED lamp (400 mW·cm^−2^) was used as a visible light source. Electrochemical impedance spectroscopy (EIS) was carried out using a potential static method in the frequency range of 0.01 to 100 kHz. The flat-band potential of the photocatalysts was derived from Mott–Schottky (MS) curves, which were received at three different frequencies of 1000, 2000, and 3000 Hz. Extrapolation of the C^−2^ curve in the M-S plot to zero gives the flat-band potential (E_fb_). For the n-type semiconductor, E_CB_ is approximately 0.1 V more negative than E_fb_ [75]. The conduction band (CB) edge position (E_CB_) against the normal hydrogen electrode (NHE) potential was calculated based on the relation:

E_CB_(NHE) = E_Ag/AgCl_ + E°_Ag/AgCl_ (E°_Ag/AgCl_ = 0.209 V) (2)

The valence band (VB) edge position (E_VB_) was calculated using E_CB_ and the band gap energy (E_g_).

E_VB_ = E_g_ + E_CB_ (3)

### 3.5. Adsorption Experiments 

For adsorption studies, 10 mg of the photocatalyst was added to 30 mL of a 2,4-D solution (20 mg∙L^−1^). The experiments were performed at 25 °C with an agitation speed of 500 rpm. At regular time intervals, the suspension was filtered through a syringe filter (0.2 μm glass fiber/PTFE membrane) and the 2,4-D concentration was analyzed by HPLC (Agilent 1260) equipped with a Kinetex C18 column (Phenomenex Inc., Torrance, CA, USA, 2.6 µm, 150 × 3 mm). The mobile phase consisted of acetonitrile (0.05 vol% TFA) and distilled water (0.15 vol% TFA) in a ratio of 30:70 *v*/*v*. Chromatographic separation was performed at 0.6 mL∙min^−1^ and a 40 °C column temperature, and a 5 μL sample volume was injected. Chromatograms were recorded at 210 and 230 nm. The adsorbed amount of 2,4-D on the materials (Q_t_) was expressed in (mg∙g^−1^) and calculated with the following equation:

(4) $$Q_{t}=\frac{(C-C_{t})V}{M}$$

where *C* is the initial concentration, *C_t_* is the concentration of 2,4-D in solution at time *t* in mg·L^−1^, *V* is the volume of the solution (L), and *M* is the weight of the adsorbent (g).

To study adsorption isotherms, experiments with BMO-400 (EG) were performed with different initial 2,4-D concentrations (with a range of 10–80 mg∙L^−1^). For all experiments, a temperature-controlled reactor (25 °C) with 30 mL of the solution and 10 mg of BMO-400 (EG) was used at an agitation speed of 500 rpm. At equilibrium time, the adsorbents were separated with a syringe filter (0.2 μm glass fiber/PTFE) and the 2,4-D filtrate was analyzed using HPLC. The equilibrium adsorption capacity of 2,4-D was calculated using the following equation:

(5) $$Q_{e}=\frac{(C-C_{e})V}{M}$$

where $Q_{e}$ (mg∙g^−1^) is the amount of adsorbed 2,4-D at equilibrium time and C and $C_{e\;}$ (mg·L^−1^) are the concentrations of 2,4-D in the solution obtained at the initial and equilibrium times, respectively. 

To explore the relationship between the adsorption species and their equilibrium concentrations, the Langmuir and Freundlich isotherm models were applied and calculated using the following equations:

(6) $$\mathrm{Langmuir}:\;\frac{C_{e}}{Q_{e}}=\frac{1}{K_{L}\cdot Q_{m}}+\frac{1}{Q_{m}}\cdot C_{e}$$

(7) Freundlich: Qe=KF·Ce1n

where $Q_{m}$ (mg·g^−1^) is the theoretical maximum monolayer capacity referred to as the amount of 2,4-D required to occupy all the available sites per unit mass of the sample, $K_{L}$ (L·mg^−1^) is the Langmuir equilibrium constant related to the affinity of binding sites with the adsorbate, $K_{F}$ (mg·g·(mg·L)^(−1/n)^) is the Freundlich constant, and (1/n) is the Freundlich intensity parameter related to the intensity of the adsorption driving force or the surface heterogeneity. 

### 3.6. Photocatalytic Degradation Experiments

#### 3.6.1. Photocatalytic 2,4-D Degradation

To study the photocatalytic degradation of 2,4-D with the synthesized Bi_2_MoO_6_ materials, 10 mg of the catalyst was added to 30 mL of the aqueous herbicide solution with the initial concentration of 20 mg∙L^−1^. The light source was a blue light LED array (SMD 3528 LEDs, 465–470 nm, LEDxON Modular GmbH, Landshut, Germany) with an intensity of 32 mW∙cm^−2^, which covered the mantle of the cylindric glass reactor with a double jacket for water cooling (Figure S1). The experiments were carried out at 25 °C. Before illumination, the solution was stirred (500 rpm) in the dark for 2 h to obtain an adsorption–desorption equilibrium between 2,4-D and the photocatalysts. During irradiation, 1.0 mL of the suspension was sampled from the reaction solution at regular time intervals, filtered, and analyzed by HPLC (see adsorption experiments). The degree of mineralization was obtained from the total organic carbon (TOC) in the final reaction solution, which was filtered (0.2 μm glass fiber/PTFE filter) and measured with a TOC analyzer (multi-N/C 3100, Analytic Jena, Jena, Germany). The degradation efficiency and TOC removal were calculated by the application of the following Equations:

(8) $$\mathrm{Degradation}\;\mathrm{efficiency}\;(\%)=\frac{C-C_{t}}{C}\times 100\%$$

(9) $$\mathrm{TOC}\;\mathrm{removal}\;(\%)=\frac{\left({TOC}_{solution\;initial}-{TOC}_{solution\;after\;reaction}\right)}{{TOC}_{solution\;initial}}\times 100\%$$

#### 3.6.2. Scavenger Experiments

Scavenger experiments were performed with the addition of isopropanol (IPA, ≥99.5%), ethylenediaminetetraacetic acid (EDTA, ≥99.0%), and 1,4-benzoquinone (BQ, ≥98.0%) to capture hydroxyl radicals (·OH), holes (h^+^), and superoxide radicals (⋅O_2_^−^), respectively. Typically, 0.15 mmol trapping agents were added to 30 mL of the 2,4-D aqueous solution (20 mg∙L^−1^) under the same conditions as the photocatalytic performance test. 

Furthermore, the identification of the hydroxyl radicals was also performed. In a typical experiment, 20 mg of the catalyst was added to 30 mL of the NaOH aqueous solution (2 × 10^−3^ M) with terephthalic acid (5 × 10^−4^ M). Although similar conditions were maintained as those described previously for the photocatalytic experiment in the small batch reactor section, 4.0 mL samples were withdrawn (at 3, 6, and 24 h) and filtered. The conditions were the same as those applied for the adsorption and photocatalytic experiments. The formation of the hydroxylated terephthalic acid compound was assessed by setting 318 nm as the excitation wavelength and recording the photoluminescence spectra in the range of 350–600 nm using a Varian Cary Eclipse Fluorescence Spectrometer (Agilent, Santa Clara, CA, USA). 

#### 3.6.3. Photocatalytic Experiment in Flow

##### Coating of the Aluminum Mesh

To produce a uniform layer on the aluminum mesh, a homogenous suspension of the catalyst precursor was required. For this, BMO-400 (EG) was ground into a fine powder and 80 mg of the solid was mixed with 4 mL EtOH. The suspension was first treated for 15 min in a laboratory ultrasonic bath and then stirred slowly for 2 h at 100 rpm. The aluminum mesh was cleaned with ethanol and heat-treated in an oven at 350 °C for 3 h in the air. For the coating process, an airbrush (0.5 mm nozzle) at an air pressure of 2 bar was applied. In a ventilated environment, the front side of the mesh was uniformly sprayed with the precursor suspension from a distance of around 20 cm, with interruptions for drying, resulting in a coating of 13.3 mg of the catalyst precursor within 10 min. Further heat treatment of the coated mesh in a static oven under air (20–400 °C/75 min, 400 °C/2 h) produced 12.5 mg of a relatively strongly bonded catalyst layer (Figure S2). 

##### Photocatalytic Flow Experiments with Recirculation

The experimental setup for the continuous flow operation had the following main components: a stirred reservoir (600 rpm) containing 40 mL of aqueous 2,4-dichlorophenoxyacetic acid (20 mg·L^−1^, 9.0 × 10^−2^ mmol·L^−1^), a micro annular gear pump (mzr-2905, HNP), a microstructured photoreactor (University Jena), a LED blue light array (the same LED type as that used for batch operation), and 1/8 inch PTFE tubing (Figure S3). 

The micro-photoreactor consists of a stainless-steel body with a reactor compartment (3.3 × 9 × 0.8 cm) and a water-cooled heat sink at the back. A screw-retained steel frame fixed a borosilicate glass window (8.8 × 3.2 × 0.6 cm) with silicone gaskets and an aluminum mesh (7.9 × 2.4 × 0.1 cm) inside the reactor body, which was used as the catalyst support. For irradiation, the LED array was attached directly to the steel frame, resulting in a distance to the mesh surface of around 1.6 cm. The aqueous reservoir solution was pumped from the bottom up through the reactor (25 °C) with a flow rate of 5 mL∙min^−1^ in circulation to the reservoir. In the regular test, the reservoir solution circulated over the catalyst mesh for 2 h first without blue light irradiation and, subsequently, for 3 or 4 h with blue light irradiation. At regular time intervals (30 min), around 200 µL of the sample liquid was taken and analyzed using an Agilent HPLC system 1200 (Agilent, Santa Clara, CA, USA). After irradiation, the reaction solution was filtered through a 0.2 µm glass fiber/PTFE filter. At the end of each experiment, the mesh was flushed with water for 30 min using a flow rate of 5 mL∙min^−1^.

## 4. Conclusions

BMO materials with different morphologies were synthesized hydrothermally using different solvents. The material synthesized in ethylene glycol and calcined at 400 °C (BMO-400 (EG)) was an effective adsorbent for 2,4-D, whose adsorption can be aptly described by the Langmuir model, indicating single-layer adsorption of 2,4-D on the hydrophilic BMO surface. Moreover, among the synthesized BMO materials, BMO-400 (EG) also showed the highest photocatalytic performance in the degradation of 2,4-D under blue light LED irradiation. The reasons for this are assumed to be the lower charge carrier recombination and improved charge carrier transfer in conjunction with the higher BET surface area. Studies focused on radical scavengers indicate that holes and superoxide radicals are active species in the conversion of 2,4-D to 2,4-DCP as the main degradation intermediate. However, both active species cannot effectively degrade the main intermediate 2,4-DCP. When H_2_O_2_ was added to the reaction mixture, the degradation of the 2,4-DCP intermediate increased significantly. The fastest mineralization of 2,4-D was observed when the BMO photocatalyst and H_2_O_2_ were used simultaneously, increasing the number of active hydroxyl radicals, as these can be formed from both the photolysis of H_2_O_2_ and its reaction with CB electrons. This work shows that BMO materials are promising adsorbents and photocatalysts for wastewater treatment under visible light irradiation.

## Figures and Tables

**Table 1:** General characteristics of BMO materials annealed in air (values in parentheses show results from hydrothermal process before annealing).

Sample	Solvent	SA (m^2^·g^−1^)	PV (cm^3^·g^−1^)	Dcryst (nm)	Band Gap (eV)
BMO-150 (EG)	EG	-	-	7.7 (6.7)	2.75 (2.79)
BMO-400 (EG)	EG	33.4	0.21	10.9 (6.7)	2.74 (2.79)
BMO-400 (2 EtOH + 1 EG)	EtOH + EG	16.0	0.15	11.5 (10.8)	2.74
BMO-400 (1 EtOH + 2 H_2_O)	EtOH + H_2_O	7.0	0.04	14.5 * (14.3 ** 10.4 ***)	2.77
BMO-400 (H_2_O)	H_2_O	16.6	0.08	16.4 (15.6)	2.78

* Diffraction reflections of Bi_2_MoO_6_ and B_14_MoO_24_ were not separated, ** Bi_2_MoO_6_, *** Bi_14_MoO_24_.

## Data Availability

Data are available from authors based on reasonable requirements from readers.

## Supplementary Materials

The following supporting information can be downloaded at: https://www.mdpi.com/article/10.3390/molecules29143255/s1, Figure S1: Reaction setup for 2,4-D degradation in small batch reactor; Figure S2: (**a**) Uncoated aluminum mesh and coated mesh (**b**) before and **c**) after heat treatment; Figure S3: (**a**) LED array and (**b**) micro-photoreactor; Figure S4: XRD powder pattern of fresh BMO synthesized using different solvent; Figure S5: SEM images of freshly synthesized samples; Figure S6: FT-IR spectra of BMO samples; Figure S7: TGA profiles; Figure S8: Nitrogen adsorption–desorption isotherms of samples annealed at 400 °C; Figure S9: Photoluminescence spectra of samples annealed at 400 °C; Figure S10: (**a**) Photocurrent responses and (**b**) EIS Nyquist plots of BMO (EG) and BMO-400 (EG); Figure S11: Mott–Schottky plots; Figure S12: Plot of adsorbed 2,4-D amount versus time on BMO synthesized in different solvents and annealed at 400 °C; Figure S13: Contact angle measurement of (**a**) BMO-400 (H_2_O), (**b**) BMO-400 (EG); Figure S14: Zeta potential of samples annealed at 400 °C as a function of pH; Figure S15: (**a**) Plot of 2,4-D concentration versus irradiation time for BMO photocatalyst synthesized in EG and after thermal annealing, (**b**) Plot of 2,4-dichlorophenol (2,4-DCP) concentration versus time (m_cat_ = 10 mg, C_(2,4-D)_ = 20 mg·L^−1^, V = 30 mL, blue LED (λ_max_ = 467 nm), T = 25 °C).; Figure S16: Photocatalytic degradation of 2,4-D for uncalcined BMO; Figure S17: The pseudo-first order fitted kinetics curves for photocatalytic degradation of 2,4-D using (**a**) freshly synthesized BMO and (**b**) BMO annealed at 400 °C; Figure S18: (**a**) Plot of 2,4-D concentration versus time in presence of different H_2_O_2_ concentrations for BMO-400 (EG) and (**b**) plot of 2,4-dichlorophenol (2,4-DCP) concentration versus irradiation time, and c) TOC removal under blue light irradiation in the presence of different concentrations of H_2_O_2_ after 3h.; Figure S19: (**a**) Plot of 2,4-D concentration and (**b**) plot of 2,4-dichlorophenol (2,4-DCP) concentration versus irradiation time using different gas atmosphere; Figure S20: Fluorescence spectra of blue light irradiated BMO-400 (EG) suspension containing terephthalic acid; Table S1: Calculated valence band (VB) and conduction band (CB) edge position of the materials; Table S2: The Langmuir and Freundlich isotherm constants for the adsorption of 2,4-D; Table S3: Comparison of maximum adsorption capacity (Q_m_) of 2,4-D using BMO-400 (EG) with that of other adsorbents; Table S4: Pseudo-first-order rate constant of 2,4-D degradation for different BMO samples under irradiation with blue LED light.; Table S5: Comparison of 2,4-D degradation rate with literature data; Table S6: Pseudo-first-order 2,4-D decay rate constant in presence of H_2_O_2_; Table S7: The elemental surface composition of different BMO samples. Refs. [76,77,78,79,80,81,82,83,84] are cited in the Supplementary Materials.
