# Supplementary material for: Effect of Bi2MoO6 Morphology on Adsorption and Visible-Light-Driven Degradation of 2,4-Dichlorophenoxyacetic Acid
Source: Molecules. 2024 Jul 10;29(14):3255. doi: 10.3390/molecules29143255 (PMC11278676; doi:10.3390/molecules29143255)
Supplement: Supplementary file 1 [file molecules-29-03255-s001.zip › molecules-3025780-supplementary.pdf]

Supplementary Material

Effect of Bi_2MoO_6 Morphology on Adsorption and Visible-Light-Driven Degradation of 2,4-Dichlorophenoxyacetic Acid

Thi Thanh Hoa Duong [1], Shuoping Ding [1], Michael Sebek [1], Henrik Lund [1], Stephan Bartling [1], Tim Peppel [1], Thanh Son Le [2] and Norbert Steinfeldt [1,*]

[1] Leibniz Institute for Catalysis e.V. (LIKAT), Albert-Einstein-Street 29a, 18059 Rostock, Germany; hoa.duong@catalysis.de (T.T.H.D.); shuoping.ding@catalysis.de (S.D.); michael.sebek@catalysis.de (M.S.); henrik.lund@catalysis.de (H.L.); stephan.bartling@catalysis.de (S.B.); tim.peppel@catalysis.de (T.P.)

[2] Faculty of Chemistry, VNU University of Science, Hanoi 100000, Vietnam; sonlt@vnu.edu.vn

* Correspondence: norbert.steinfeldt@catalysis.de; Tel.: +49-(381)-1281-319

1. Figures section

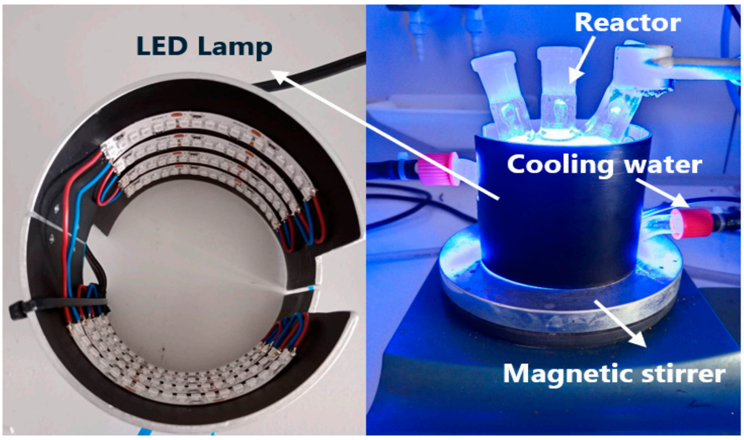

Figure S 1. Reaction set up for 2,4-D degradation in small batch reactor (blue light LED intensity: 32 mW·cm^{-2}).

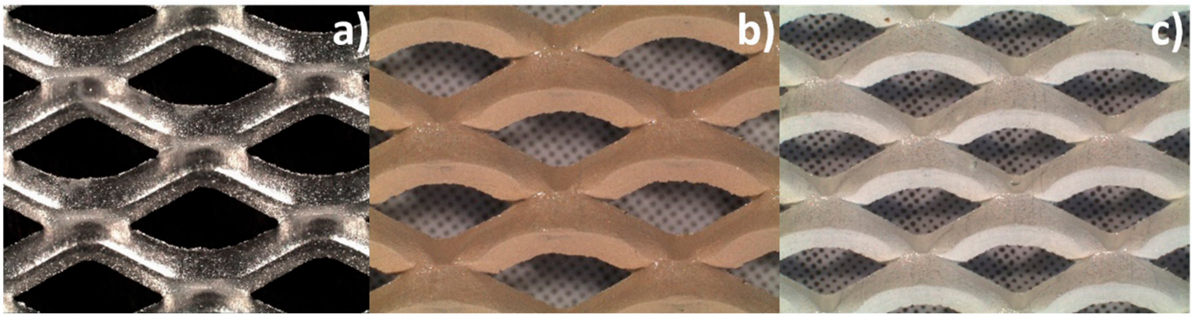

Figure S 2. a) Uncoated aluminum mesh, **b)** the coated mesh before, and **c)** after heat-treatment.

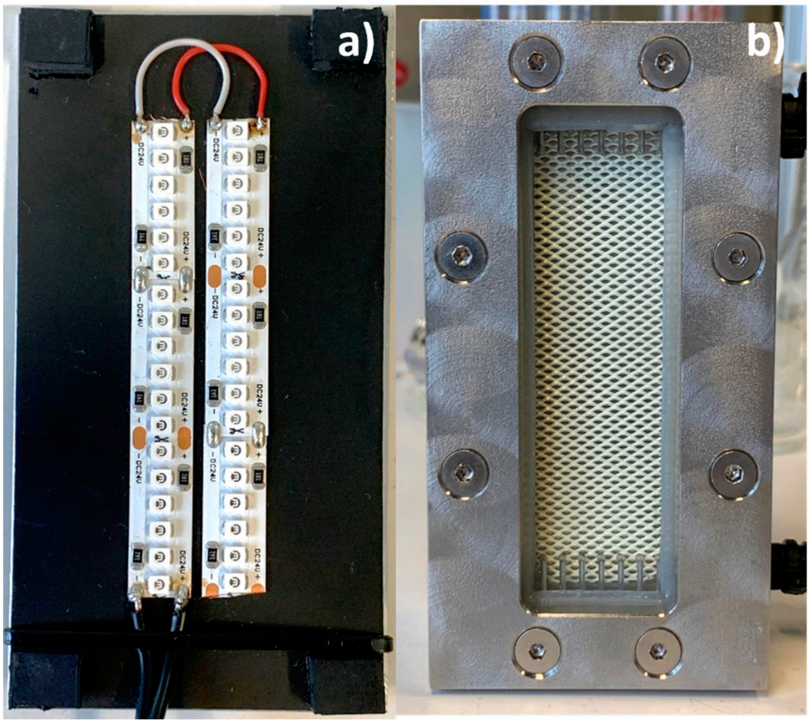

Figure S 3. a) Led array and **b)** micro-photoreactor.

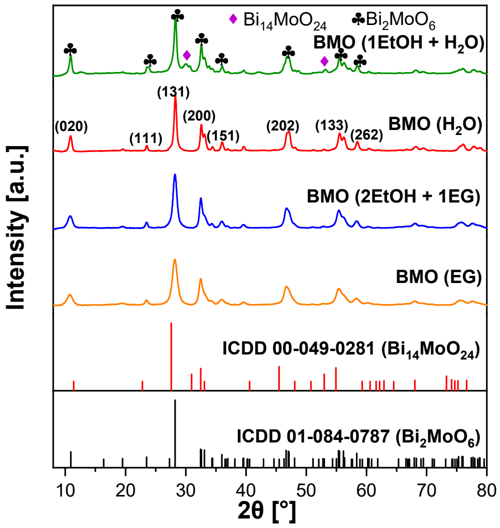

Figure S 4. XRD powder pattern of freshly BMO synthesized using different solvent.

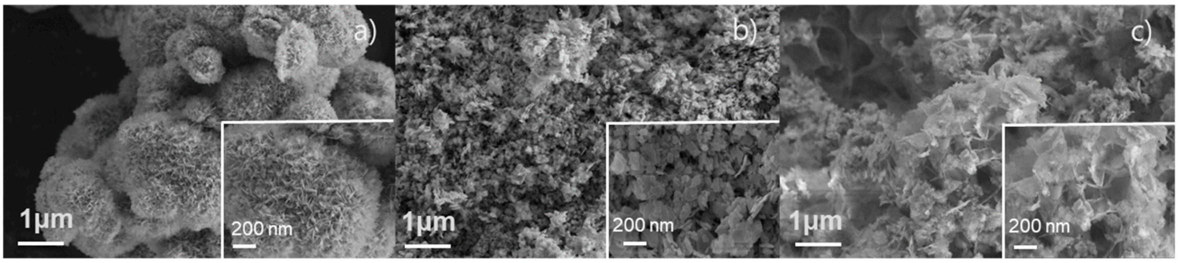

Figure S 5. SEM images of the sample **a**) BMO (2 EtOH + 1 EG), **b**) BMO (H_2O), and **c**) BMO (1 EtOH + 2 H_2O) after hydrothermal process and before thermal treatment.

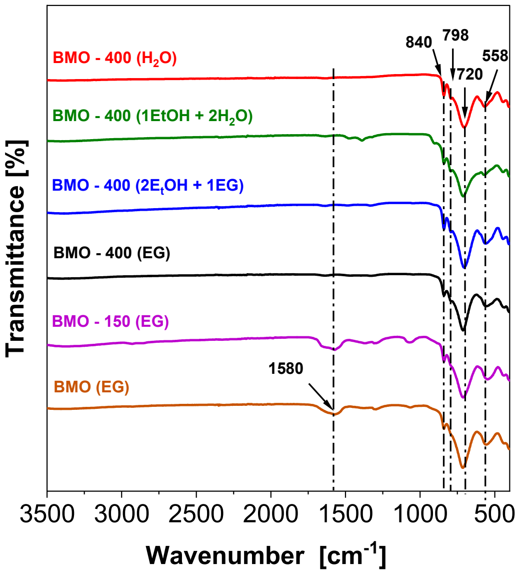

Figure S 6. FT-IR spectra of BMO samples.

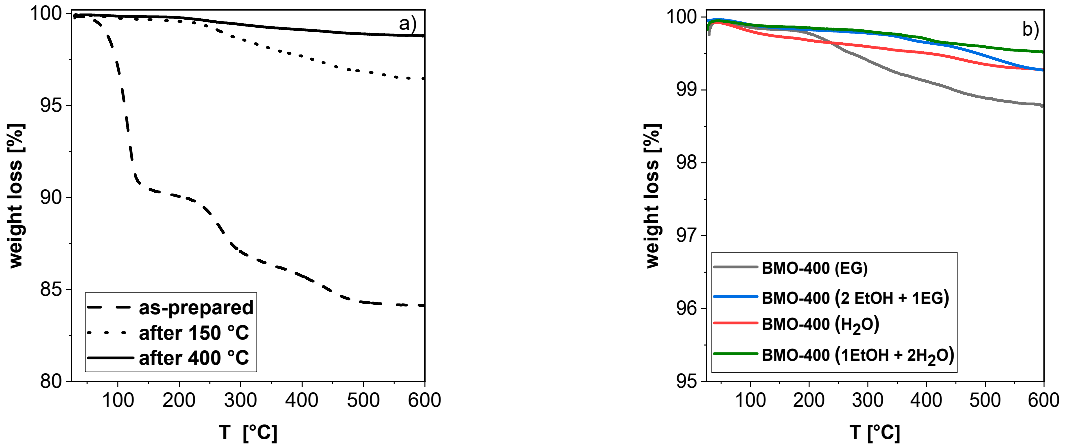

Figure S 7. TGA profile of the samples **a)** BMO (EG), BMO-150 (EG), BMO-400 (EG), and **b)** after thermal annealing at 400 °C.

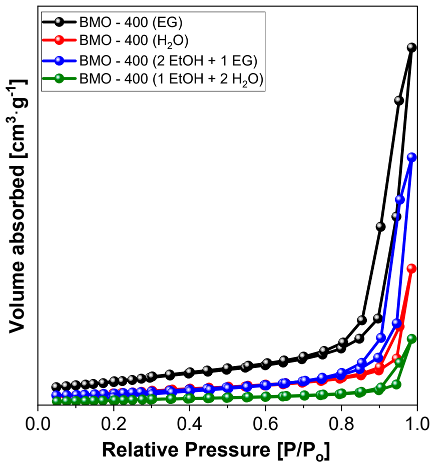

Figure S 8. Nitrogen adsorption – desorption isotherms of samples annealed at 400 °C.

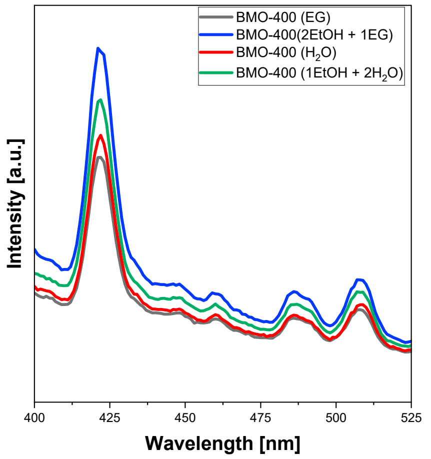

Figure S 9. Photoluminescence spectra of samples annealed at 400 °C.

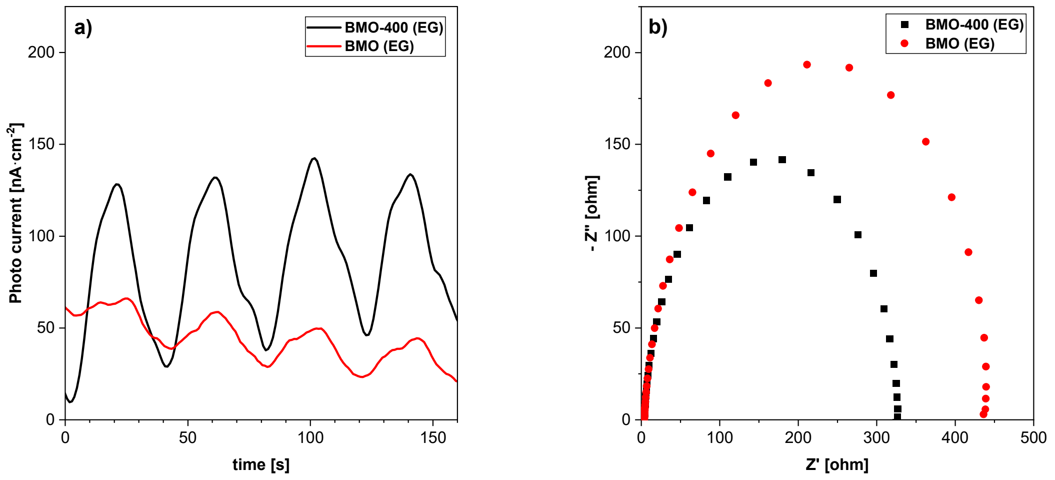

Figure S 10. a) Photocurrent responses and **b)** EIS Nyquist plots of BMO (EG) and BMO-400 (EG) samples obtained under irradiation with visible light (λ = 430 nm).

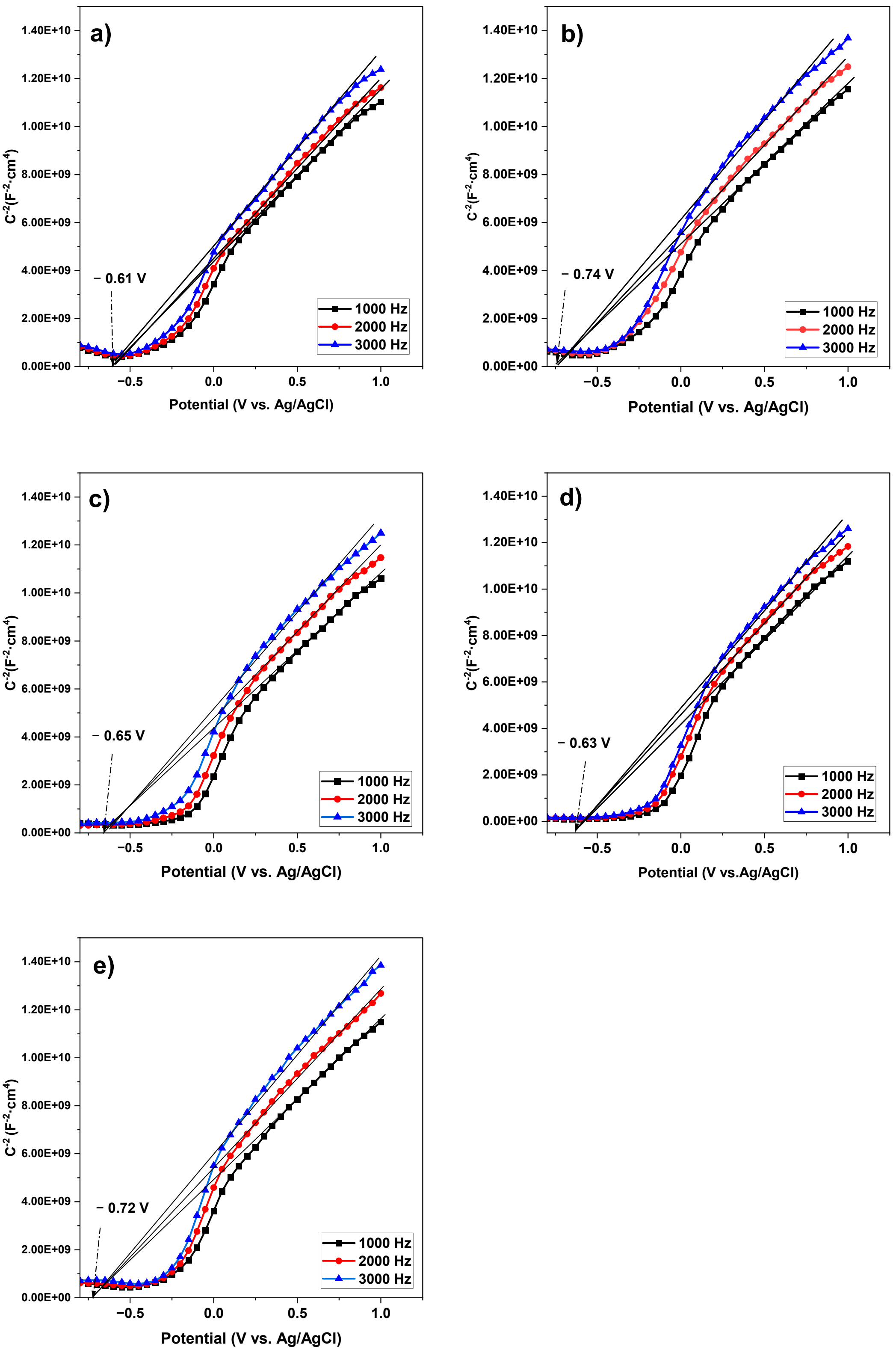

Figure S 11. Mott -Schottky plot of **a)** BMO-400 (EG), **b)** BMO-400 (2 EtOH + 1 EG), **c)** BMO-400 (H_2O), **d)** BMO-400 (1 EtOH + 2 H_2O), and **e)** BMO (EG).

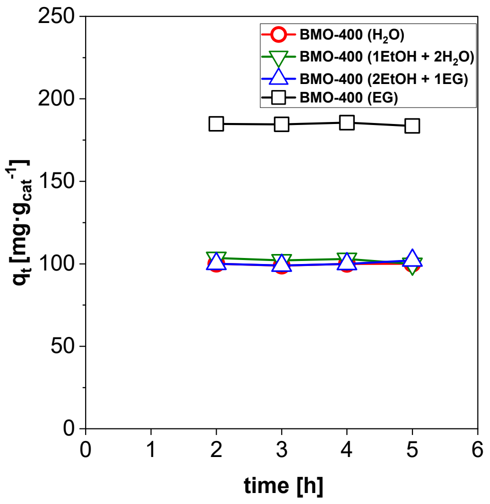

Figure S 12. Plot of adsorbed 2,4-D amount versus time on BMO synthesized in different solvents and annealed at 400 °C ($C_{(2,4\text{-}D)}$ =20 mg·L^{-1}, m_{cat} = 10 mg, V = 30 mL).

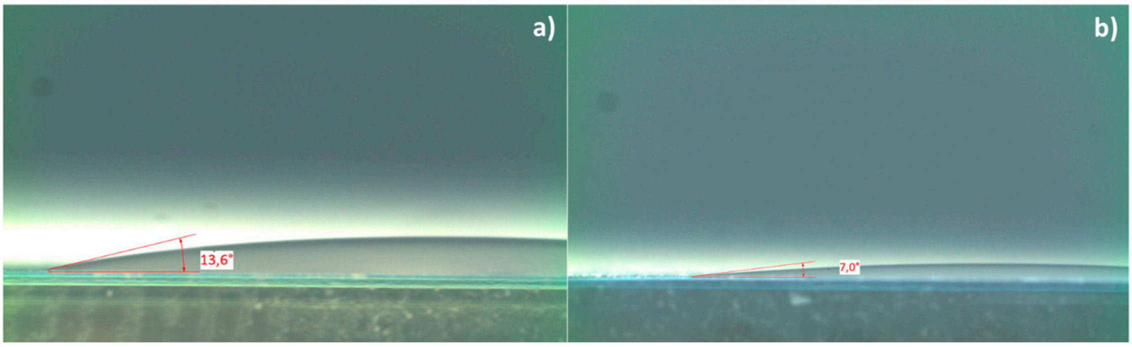

Figure S 13. Contact angle of **a)** BMO-400 (H_2O), **b)** BMO-400 (EG).

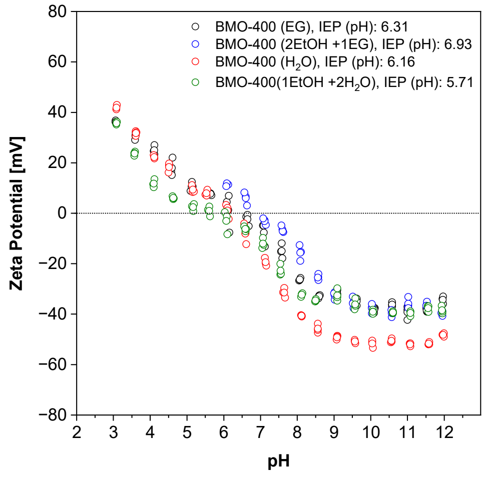

Figure S 14. Zeta potential of samples annealed at 400 °C as a function of pH.

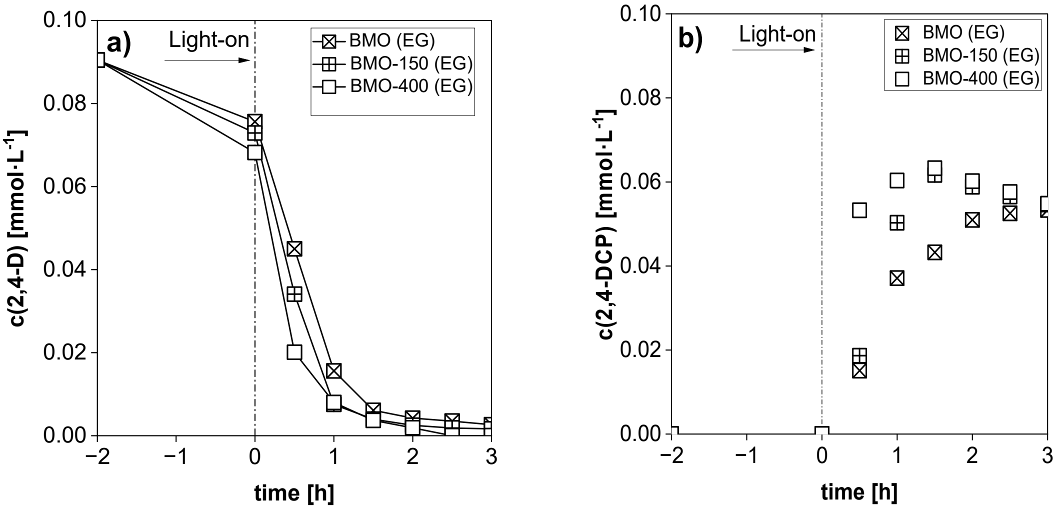

Figure S 15. a) Plot of 2,4-D concentration versus irradiation time for BMO photocatalyst synthesized in EG and after thermal annealing, **b)** Plot of 2,4-dichlorophenol (2,4-DCP) concentration versus time (m_{cat} = 10 mg, $C_{(2,4\text{-}D)}$ = 20 mg·L^{-1}, V = 30 mL, blue LED (λ_{max} = 467 nm), T = 25°C).

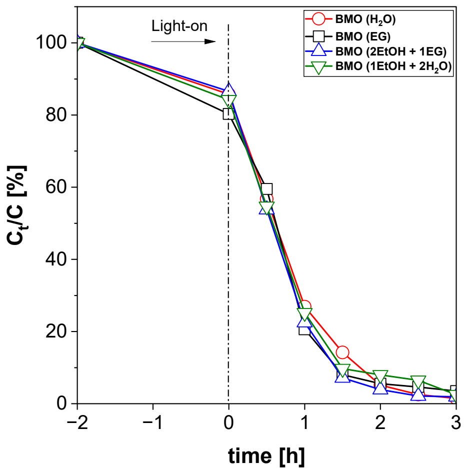

Figure S 16. Photocatalytic degradation of 2,4-D for BMO synthesized using different solvents (m_{cat} = 10 mg, $C_{(2,4\text{-}D)}$ = 20 mg·L^{-1}, V = 30 mL, blue LED (λ_{max} = 467 nm), T = 25°C).

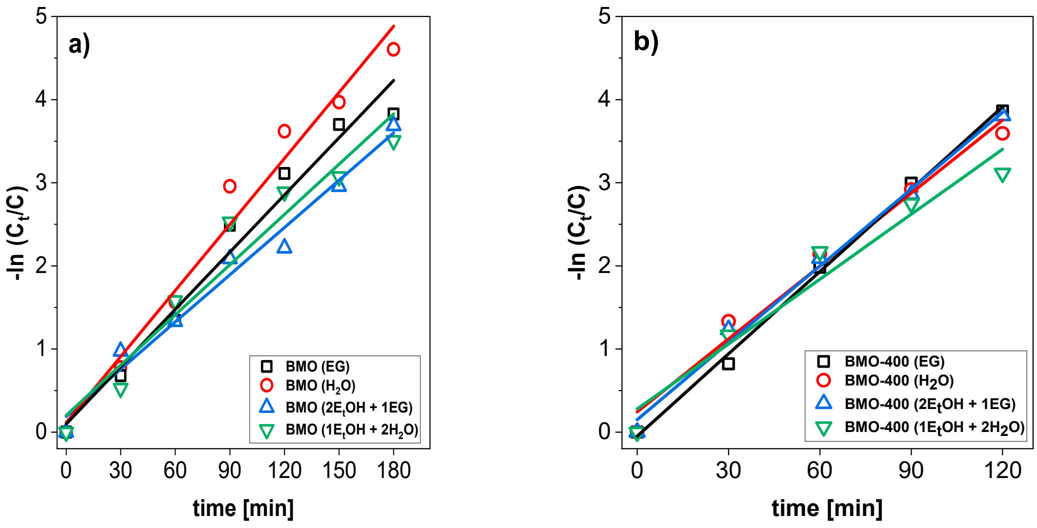

Figure S 17. The pseudo-first order fitted kinetics curves for photocatalytic degradation of 2,4-D using **a)** freshly synthesized BMO and **b)** BMO annealed at 400 °C.

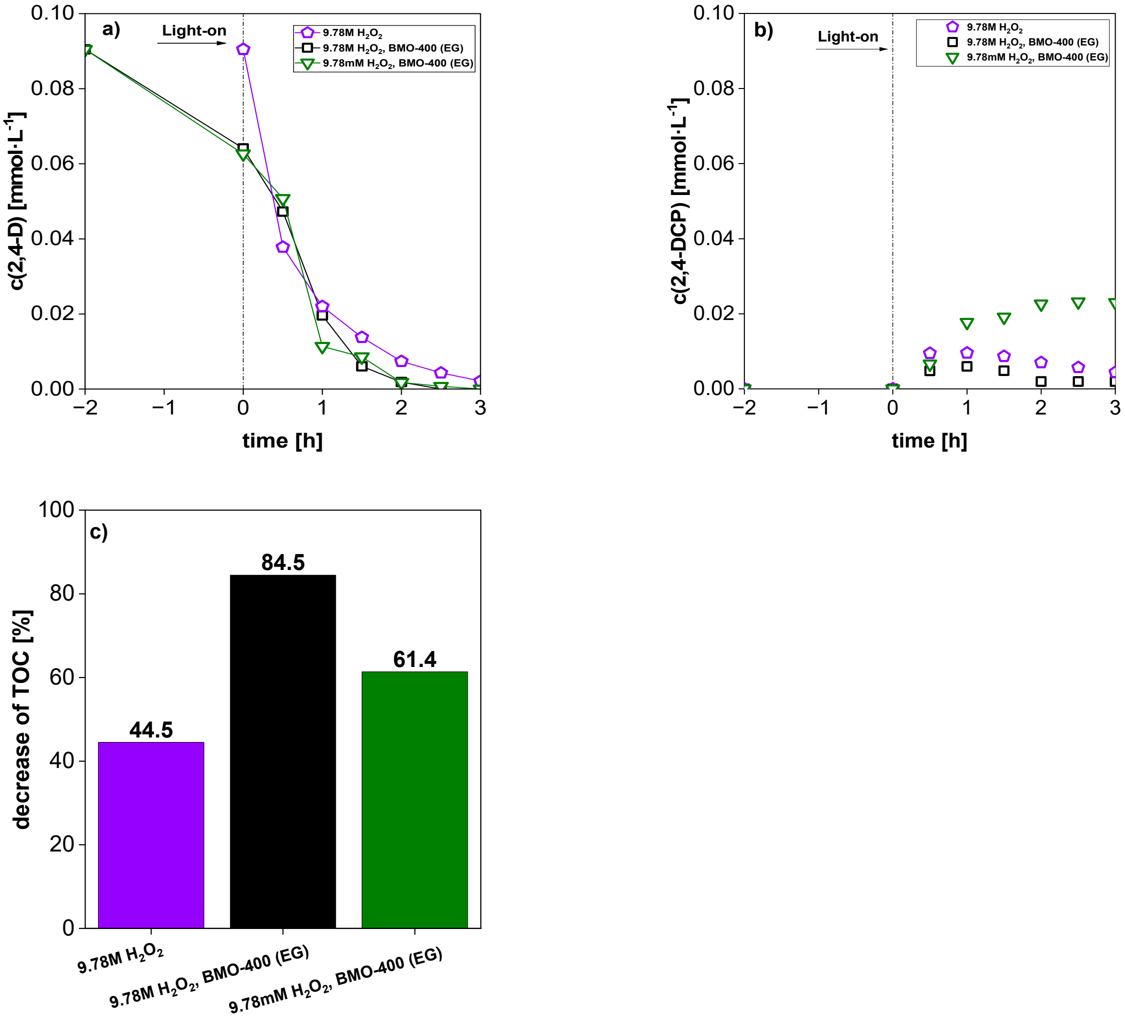

Figure S 18. **a)** Plot of 2,4-D concentration versus time in presence of different H_2O_2 concentration for BMO-400 (EG) and **b)** plot of 2,4-dichlorophenol (2,4-DCP) concentration versus irradiation time (m_{cat} = 10 mg, $C_{(2,4\text{-}D)}$ = 20 mg·L^{-1}, V = 30 mL, blue LED (λ_{max} = 467 nm), T = 25°C, V_{H2O2}=1 mL), and **c)** TOC removal under blue light irradiation in the presence of different concentration of H_2O_2 after 3h.

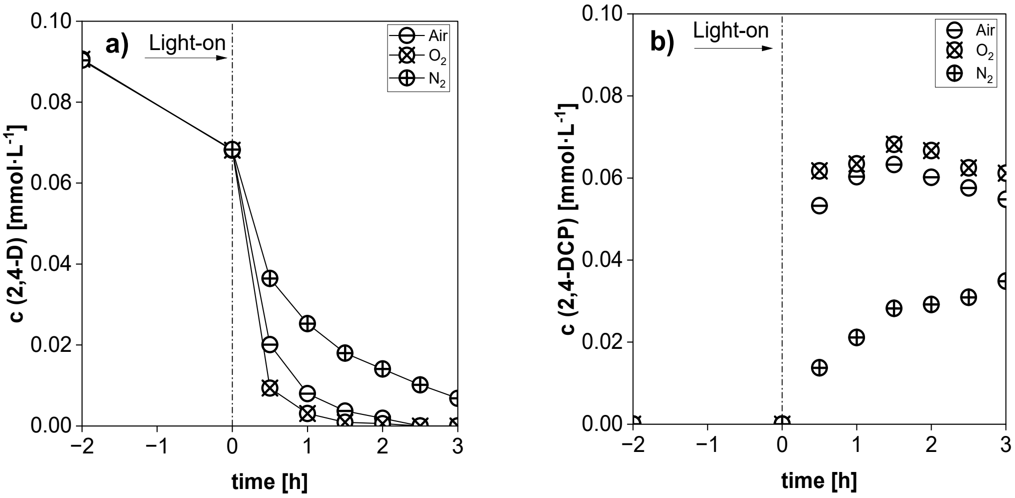

Figure S 19. a) Plot of 2,4-D concentration and **b)** plot of 2,4-dichlorophenol (2,4-DCP) concentration versus irradiation time using different gas atmosphere (m_{cat} = 10 mg, $C_{(2,4-D)}$ = 20 mg·L^{-1}, V = 30 mL, blue LED (λ_{max} = 467 nm), T = 25°C).

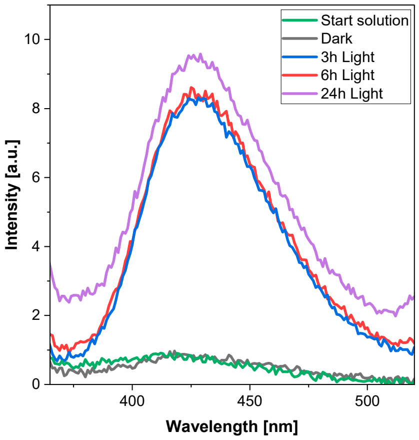

Figure S 20. Fluorescence spectra of blue light irradiated BMO-400 (EG) suspension in terephthalic acid at different irradiation times.

2. Tables section

Table S1. Calculated valence band (VB) and conduction band (CB) edge position of the materials.

Materials	E_{flat} (V, vs. Ag/AgCl)	E_{CB} (V, vs. NHE)	E_{VB} (V, vs. NHE)
BMO (EG)	– 0.72	– 0.61	2.18
BMO – 400 (EG)	– 0.61	– 0.50	2.24
BMO – 400 (H_2O)	– 0.65	– 0.54	2.24
BMO – 400 (2 EtOH + 1 EG)	– 0.74	– 0.63	2.11
BMO – 400 (1 EtOH + 2 H_2O)	– 0.63	– 0.52	2.25

Table S2. The Langmuir and Freundlich isotherm constant for the adsorption of 2,4-D. The parentheses include standard errors.

Adsorbent	Langmuir	Freundlich

	K_L	Q_m	R^2	K_F	n	R^2
BMO – 400 (EG)	0.125	320 (13.40)	0.988	94.60	3.68	0.984

Table S3. Comparison of maximum adsorption capacity (Q_m) of 2,4-D using different adsorbents.

**Adsorbents**	**Q_m (mg·g_{cat}^{-1})**	**References**
Mesoporous Carbon CKIT-6	109.0	[1]
Corn cob biochar	37.4	[2]
Magnetic Fe_3O_4@graphene nanocomposite	32.31	[3]
Magnetic activated charcoal/Fe_2O_3 nanocomposite	255.1	[4]
MIEX Resin	293.0	[5]
BMO – 400 (EG)	320.0	**This study**

Table S4. Pseudo-first-order rate constant of 2,4-D degradation for synthesized BMO samples under irradiation with blue LED light.

**Materials**	**k (min^{-1})**	**R^2**
BMO (EG)	0.023	0.965
BMO (H_2O)	0.026	0.969
BMO (2 EtOH + 1 EG)	0.019	0.975
BMO (1 EtOH + 2 H_2O)	0.020	0.933
BMO – 400 (EG)	0.029	0.974
BMO – 400 (H_2O)	0.033	0.996
BMO – 400 (2 EtOH + 1 EG)	0.031	0.999
BMO – 400 (1 EtOH + 2 H_2O)	0.026	0.938

Table S5. Experimental conditions and degradation rates of 2,4-D using different photocatalysts.

**Photocatalyst**	**Reaction condition**	**k (min^{-1})**	**References**
$WO_3/NaNbO_3$	[2,4-D] = 10 mg·L^{-1}, [Cat.] = 1g·L^{-1}, 50 W LED (λ = 350 ~ 650 nm)	0.0077	[6]
Curcuma longa/$BiVO_4$	[2,4-D] = 20 mg·L^{-1}, [Cat.] = 1g·L^{-1}, 45 W LED	0.0182	[7]
$ZnIn_2S_4/gC_3N_4$	[2,4-D] = 100 mg·L^{-1}, [Cat.] = 0.4 g·L^{-1}, 500 W Xe lamp (λ > 420 nm)	0.0129	[8]
Ag/$BiVO_4$	[2,4-D] = 20 mg·L^{-1}, [Cat.] = 1g·L^{-1}, Cree LED light (20 mW cm^{-2}, λ=470 nm)	0.0101	[9]
Ag_3PO_4/TiO_2	[2,4-D] = 20 mg·L^{-1}, [Cat.] = 1g·L^{-1}, 300 W Xenon	0.028	[10]

	lamp (ozone free, UV cut off filter)		
BMO – 400 (EG)	[2,4-D] = 20 mg·L^{-1}, [Cat.] = 0.33 g·L^{-1}, Blue light LED (λ = 467 nm)	0.029	**This study**

Table S6. Pseudo-first-order rate constants for 2,4-D decay in presence of H_2O_2 (9.78M).

**Materials**	**k (min^{-1})**	**R^2**
BMO – 400 (EG)/H_2O_2	0.030	0.948
BMO – 400 (H_2O)/H_2O_2	0.029	0.989
BMO – 400 (2 EtOH + 1 EG)/H_2O_2	0.028	0.963
BMO – 400 (1 EtOH + 2 H_2O)/H_2O_2	0.026	0.971
BMO – 400 (EG)/H_2O_2*	0.031	0.956
Pure H_2O_2	0.019	0.991

*concentration of H_2O_2: 9.78 mM

Table S7. The content (at.%) of element in the materials.

**Materials**	**Bi (at.%)**	**Mo (at.%)**	**C (at.%)**	**O (at.%)**
BMO – 400 (EG)	25.22	7.90	18.17	47.31
BMO – 400 (EG)$^{(a)}$	21.24	5.58	25.45	32.55
BMO – 400 (EG)$^{(b)}$	17.0	4.88	35.81	29.16
BMO-400 (H_2O)	27.06	9.68	12.45	49.50
BMO-400 (2 EtOH + 1 EG)	25.69	9.21	9.99	50.62
BMO-400 (1 EtOH + 2 H_2O)	24.90	9.76	10.04	48.84

(a) After 3rd run reaction

(b) After 24 h reaction

References

1. Goscianska, J.; Olejnik, A., Removal of 2, 4-D herbicide from aqueous solution by aminosilane-grafted mesoporous carbons. *Adsorption* **2019,** 25, (3), 345-355.
2. Binh, Q. A.; Nguyen, H.-H., Investigation the isotherm and kinetics of adsorption mechanism of herbicide 2, 4-dichlorophenoxyacetic acid (2, 4-D) on corn cob biochar. *Bioresour. Technol.* **2020,** 11, 100520.
3. Liu, W.; Yang, Q.; Yang, Z.; Wang, W., Adsorption of 2, 4-D on magnetic graphene and mechanism study. *Colloids Surf. A Physicochem. Eng. Asp.* **2016,** 509, 367-375.
4. Vinayagam, R.; Pai, S.; Murugesan, G.; Varadavenkatesan, T.; Narayanasamy, S.; Selvaraj, R., Magnetic activated charcoal/Fe_2O_3 nanocomposite for the adsorptive removal of 2, 4-Dichlorophenoxyacetic acid (2,4-D) from aqueous solutions: synthesis, characterization, optimization, kinetic and isotherm studies. *Chemosphere* **2022,** 286, 131938.
5. Ding, L.; Lu, X.; Deng, H.; Zhang, X., Adsorptive removal of 2, 4-dichlorophenoxyacetic acid (2, 4-D) from aqueous solutions using MIEX resin. *Ind. Eng. Chem. Res.* **2012,** 51, (34), 11226-11235.
6. Hernández-Moreno, E.; de la Cruz, A. M.; Hinojosa-Reyes, L.; Guzmán-Mar, J.; Gracia-Pinilla,

M.; Hernández-Ramírez, A., Synthesis, characterization, and visible light–induced photocatalytic evaluation of WO_3/$NaNbO_3$ composites for the degradation of 2, 4-D herbicide. *Mater. Today Chem.* **2021,** 19, 100406.
7. Chawla, H.; Garg, S.; Rohilla, J.; Szamosvölgyi, Á.; Efremova, A.; Szenti, I.; Ingole, P. P.; Sápi, A.; Kónya, Z.; Chandra, A., Visible LED-light driven photocatalytic degradation of organochlorine pesticides (2, 4-D & 2, 4-DP) by Curcuma longa mediated bismuth vanadate. *J. Clean. Prod.* **2022,** 367, 132923.
8. Qiu, P.; Yao, J.; Chen, H.; Jiang, F.; Xie, X., Enhanced visible-light photocatalytic decomposition of 2, 4-dichlorophenoxyacetic acid over $ZnIn_2S_4$/g-C_3N_4 photocatalyst. *J. Hazard. Mater.* **2016,** 317, 158-168.
9. Sánchez, O. A.; Rodríguez, J. L.; Barrera-Andrade, J. M.; Borja-Urby, R.; Valenzuela, M. A., High performance of Ag/$BiVO_4$ photocatalyst for 2, 4-Dichlorophenoxyacetic acid degradation under visible light. *Appl. Catal. A: Gen.* **2020,** 600, 117625.
10. Amiri, F.; Dehghani, M.; Amiri, Z.; Yousefinejad, S.; Azhdarpoor, A., Photocatalytic degradation of 2, 4-dichlorophenoxyacetic acid from aqueous solutions by Ag_3PO_4/TiO_2 nanoparticles under visible light: kinetic and thermodynamic studies. *Water Science and Technology* **2021,** 83, (12), 3110-3122.
